# Laparoscopic versus open parenchymal preserving liver resections in the posterosuperior segments (ORANGE Segments): a multicentre, single-blind, randomised controlled trial

**DOI:** 10.1016/j.lanepe.2025.101228

**Published:** 2025-02-20

**Authors:** Jasper P. Sijberden, Christoph Kuemmerli, Francesca Ratti, Mathieu D'Hondt, Robert P. Sutcliffe, Roberto I. Troisi, Mikhail Efanov, Robert S. Fichtinger, Rafael Díaz-Nieto, Giuseppe M. Ettorre, Aali J. Sheen, Krishna V. Menon, Marc G. Besselink, Zahir Soonawalla, Somaiah Aroori, Rebecca Marino, Celine De Meyere, Ravi Marudanayagam, Giuseppe Zimmitti, Bram Olij, Zina Eminton, Lloyd Brandts, Clarissa Ferrari, Ronald M. van Dam, Luca A. Aldrighetti, Siân Pugh, John N. Primrose, Mohammed Abu Hilal

**Affiliations:** a Department of Surgery, Fondazione Poliambulanza Istituto Ospedaliero, Brescia, Italy; b Amsterdam UMC Location University of Amsterdam, Department of Surgery, Amsterdam, the Netherlands; c Cancer Center Amsterdam, Amsterdam, the Netherlands; d Hepatobiliary and Pancreatic Surgical Unit, University Hospital Southampton NHS Foundation Trust, Southampton, United Kingdom; e Department of Surgery, Clarunis University Digestive Health Care Center Basel, University Hospital Basel, Basel; f Vita-Salute San Raffaele University, Milano, Italy; g Hepatobiliary Surgery Division, IRCCS San Raffaele Hospital, Milan, Italy; h Department of Digestive and Hepatobiliary/Pancreatic Surgery, Groeninge Hospital, Kortrijk, Belgium; i Department of Hepatobiliary and Pancreatic Surgery, University Hospitals Birmingham NHS Foundation Trust, Birmingham, United Kingdom; j Division of HPB, Minimally Invasive and Robotic Surgery, Transplantation Center, Department of Clinical Medicine and Surgery, Federico II University Hospital, Naples, Italy; k Department of Human Structure and Repair, Ghent University, Ghent, Belgium; l Department of Hepato-Pancreato-Biliary Surgery, Moscow Clinical Research Centre, Moscow, Russia; m Department of Surgery, Maastricht University Medical Centre+, Maastricht, the Netherlands; n Department of General, Visceral and Transplant Surgery, University Hospital RWTH Aachen, Aachen, Germany; o Hepatobiliary Surgery Unit, Aintree University Hospital, Liverpool, United Kingdom; p Division of General Surgery and Liver Transplantation, San Camillo Hospital, Rome, Italy; q Department of Surgery, Manchester University Foundation Trust, Manchester, United Kingdom; r Institute of Liver Studies, King's College Hospital, London, United Kingdom; s Department of Surgery, Oxford University Hospital NHS Foundation Trust, Oxford, United Kingdom; t Peninsula HPB Unit, Derriford Hospital, University Hospitals Plymouth NHS Trust, Plymouth, United Kingdom; u GROW School for Oncology & Reproduction, Maastricht University, Maastricht, the Netherlands; v Southampton Clinical Trials Unit, University of Southampton, Southampton, United Kingdom; w Department of Clinical Epidemiology and Medical Technology Assessment, Maastricht University Medical Centre+, Maastricht, the Netherlands; x Research and Clinical Trials Unit, Fondazione Poliambulanza Istituto Ospedaliero, Brescia, Italy; y Department of Oncology, Addenbrooke's Hospital, Cambridge, United Kingdom; z University Surgery and Perioperative and Critical Care Theme, NIHR Southampton Biomedical Research Centre, University Hospital Southampton/University of Southampton, Southampton, United Kingdom; aa Department of Surgery, School of Medicine, The University of Jordan, Amman, Jordan

**Keywords:** Liver neoplasms, Hepatectomy, Laparoscopic liver resection, Open liver resection

## Abstract

**Background:**

An increasing number of liver resections are performed laparoscopically, while laparoscopic resection of lesions in the posterosuperior segments is technically challenging. We aimed to assess the outcomes of laparoscopic and open parenchymal preserving resection of lesions in the posterosuperior segments in a randomised controlled trial.

**Methods:**

In this multicentre, patient-blinded, superiority randomised controlled trial, patients requiring parenchymal preserving liver resection for tumours in segment 4a, 7, or 8 were enrolled at 17 centres and randomised 1:1 to laparoscopic or open surgery using a minimisation scheme stratifying for centre and lesion size. The primary endpoint was time to functional recovery measured in postoperative days. To detect a difference in time to functional recovery of two days the sample size needed 250 patients, an interim analysis was planned with 125 patients. Patients and outcome assessors were blinded to the allocation. The study was registered on clinicaltrials.gov, NCT03270917.

**Findings:**

Between November 2017 and November 2021, 251 patients were randomised to laparoscopic (n = 125) or open (n = 126) surgery. The majority of patients had a preoperative diagnosis of cancer (225/246 = 91.5%). Time to functional recovery was 3 days (IQR 3–5) in the laparoscopic group compared to 4 days (IQR 3–5) in the open group (difference −19.2%, 96% CI −28.8% to −8.4%; p < 0.001). Hospital stay was similarly shorter in the laparoscopic group (4 days, IQR 3–5 versus 5 days, IQR 4–7; p < 0.001). There were three deaths in the laparoscopic group (3/122 = 2.5%) and one in the open group (1/124 = 0.8%) within 90 days of resection (p = 0.336). Overall postoperative morbidity, severe morbidity, liver-specific morbidity, and readmission were not statistically significant different between the groups. The radical resection (R0) rate in patients with cancer was comparable (laparoscopic 93/106 = 87.7% versus open 97/113 = 85.8%, p = 0.539).

**Interpretation:**

For patients with lesions in the posterosuperior segments of the liver, laparoscopic surgery, as compared to open surgery, reduces time to functional recovery. However, this reduction in time to functional recovery did not meet the hypothesized difference in time to functional recovery of two days.

**Funding:**

This investigator-initiated trial was funded by 10.13039/100009933Ethicon (Johnson & Johnson), Cancer Research United Kingdom, and Maastricht University Medical Centre+.

**Research in context**

**Evidence before this study**

An extensive literature search was conducted using PubMed, Embase and the Cochrane library for studies published from the inception of these databases until August 24, 2023. The search terms “hepatectomy”, “laparoscopy” and “posterosuperior segment” and synonyms were used. Studies in any language reporting comparative outcomes of laparoscopic and open surgery were included. In the most recently published systematic review found, 11 retrospective studies with a total of 1023 patients were included. A meta-analysis of these studies associated laparoscopic surgery with less blood loss (mean difference −114.71 ml, 95% CI −165.64 to −63.79, p < 0.001), a lower postoperative morbidity rate (odds ratio 0.45, 95% CI 0.33–0.61, p < 0.001), severe postoperative morbidity rate (odds ratio 0.52, 95% CI 0.36–0.73, p < 0.001) and shorter length of hospital stay (mean difference −2.01 days, 95% −2.09 to −1.92, p < 0.001) but a longer operative time (mean difference 50.28 min, 95% CI 22.29–78.27, p < 0.0001) with comparable long-term survival outcomes when compared to open surgery. However, in this meta-analysis significant heterogeneity was noted in several of the reported outcomes, all included studies had a high risk of bias in participant selection and the largest included study only comprised 172 patients. No data concerning time to functional recovery was reported.

**Added value of this study**

To the best of our knowledge, this is the first randomised controlled trial which compares the outcomes of the laparoscopic and open approach when adopted to perform parenchymal preserving liver resections in the posterosuperior segments of the liver. This trial provides the highest level of evidence in the field, demonstrating that the laparoscopic approach can reduce time to functional recovery and hospital stay, even when adopted for these technically complex resections. Furthermore, it does not seem to have an adverse effect on patient-reported outcomes, health resource costs or oncological outcomes.

**Implications of all the available evidence**

Based on the results of this study, it is expected that guidelines will mention the laparoscopic approach as a viable option for resections in the posterosuperior segments when performed in centres with the appropriate expertise.

## Introduction

Surgical resection is the cornerstone of curative treatment for both primary and metastatic cancer of the liver. The main indication for liver resection in Western countries is colorectal liver metastasis (CRLM) with reported five-year survival rates of 20–45%. The laparoscopic approach to liver resection has increasingly been adopted following evidence of efficacy in other settings.^1^ This practice is further supported by observational studies demonstrating improved perioperative and at least comparable oncological outcomes when laparoscopic surgery is used.^2^^,^^3^

Parenchymal preserving resections are increasingly preferred to minimise the risk of postoperative liver failure and preserve liver parenchyma. Lesions situated in the posterosuperior segments of the liver (Segment 1, 4a, 7 and 8) were initially considered unsuitable for the laparoscopic approach due to the difficulty in gaining adequate access to this location, limited visualization and difficulties in bleeding control.^4^ In fact, concerns were expressed that in these situations laparoscopic surgeons may unjustly shift from parenchymal preserving resections to major resections (e.g., hemi-hepatectomy) as these could be technically less challenging when performed laparoscopically.^5^

Improvements in technology and refinements of surgical techniques have resulted in an increased adoption of laparoscopic surgery to perform these procedures, although robust evidence on the safety, feasibility and oncological efficacy of laparoscopy in this setting is still lacking.^1^^,^^6^, ^7^, ^8^, ^9^, ^10^, ^11^ Given the unexpected results observed in other settings, it is crucial that the potential benefits of the laparoscopic approach to parenchymal preserving resections of lesions in the posterosuperior segments of the liver are appropriately evaluated in a randomised controlled trial.^12^ Therefore, the aim of this study was to assess the outcomes of laparoscopic and open parenchymal preserving resection of lesions in the posterosuperior segments in a randomised controlled trial.

## Methods

### Study design and participants

This multicentre, patient-blinded, randomised controlled superiority trial was conducted in 17 hepatobiliary centres from five European countries with suitable experience in open and laparoscopic liver surgery, and an implemented enhanced recovery program (Supplementary Material). Patients with a body mass index (BMI) between 18 and 35 kg/m^2^ requiring, as judged by a multidisciplinary team, a parenchymal-sparing liver resection involving one or two of segments 4a, 7, 8 or a segment 6/7 were eligible for inclusion. Patients with liver lesions too close to vascular or biliary structures to be operated laparoscopically, or who had previously undergone a liver resection were excluded. Trial candidates meeting these criteria were informed about the study by their treating surgeon in the outpatient clinic. After consenting to be further informed patients were approached and informed by the principal investigator, an authorised researcher, informed surgical resident or research nurse. After a period of reflection, patients were asked to decide whether they would participate in the trial or not. If a patient decided to participate, personal written informed consent was obtained by the principal investigator, an authorized researcher or a research nurse.

The study protocol was approved by an ethical committee and the institutional review boards at each participating centre and is reported in more detail in a previous publication.^13^ Each study participant provided written informed consent, and their data was consistently handled in pseudonymized manner. Data were reviewed by an independent data and safety monitoring board (DSMB). The study was registered on clinicaltrials.gov (NCT03270917).

### Procedures

Participants were randomly allocated in a 1:1 ratio using a minimisation scheme stratifying for centre and lesion size (<3 cm or ≥3 cm), to laparoscopic or open surgery by a designated research team member using a centralised online randomisation service.^14^ Patients and ward personnel were blinded using a large abdominal dressing until postoperative day four, unless patients had already reached the primary endpoint or their condition necessitated unblinding at an earlier stage. Operating room personnel could not be blinded due to the nature of the surgical procedures performed, but they were instructed to not disclose information regarding the surgical approach used to patients or ward personnel. Blinding efficacy was assessed by asking the patient, before removing their abdominal dressing, which approach was used to perform their surgery. Perioperative care was standardized in line with the enhanced recovery after surgery (ERAS) guidelines.^15^ Surgical techniques were not standardised and performed at the discretion of the operating surgeon.

### Primary endpoint

The primary endpoint was time to functional recovery, measured in terms of postoperative days. A patient was deemed functionally recovered when the following criteria were met: adequate pain control with oral analgesia, independently mobile at the preoperative level, tolerating solid food for at least 24 h, independent of intravenous fluid administration, and normal or normalizing liver function tests, bilirubin and coagulation studies. These criteria were based on several published randomised controlled trials in the field of pancreatic surgery.^16^, ^17^, ^18^ The criteria were adapted for liver surgery by the ORANGE study steering committee. Trained research staff (dedicated PhD students, research nurses, ward doctors) assessed if a patient met any of these criteria once per day, until the patient met all criteria and was therefore functionally recovered.

### Secondary endpoints

Secondary endpoints included operative time, blood loss, postoperative length of hospital stay, 90-day readmissions, (liver-specific) morbidity and mortality. Secondary oncological outcomes for patients with cancer comprised resection margin status, time to initiation of adjuvant systemic therapy, and recurrence-free and overall survival. The resection margin was considered microscopically tumour-free (R0) when equal to or greater than one millimetre. Recurrence-free and overall survival were defined as the interval, in months, between the date of surgery and the date when there was clinical evidence of disease recurrence or the date of death, respectively. Long-term follow-up of patients that underwent surgery for cancer was not standardized within the study, but generally patients were followed-up with imaging every 3–4 months in the first postprocedural year and every 4–6 months in the second postprocedural year. Further secondary outcome definitions and the extent of missing data is reported in the Supplementary Material.

### Sample size

To detect a difference in time to functional recovery of two days, a difference chosen based on differences in length of stay reported in the literature, the sample size needed 250 patients. This assumed a standard deviation of five days, a drop-out rate of 10%, the loss of some degrees of freedom for covariate effect estimation (centre and lesion size), a two-tailed alpha level of 0.04 and a power of 80%. Alpha was adjusted from 0.05 to 0.04 in view of multiple comparisons due to the planned interim analysis at a two-tailed alpha of 0.01. This blinded interim analysis, overseen by the independent DSMB, was carried out after 125 patients were randomised. The trial was continued as the stopping rules were not met. More details regarding the sample size calculation and interim analysis are available in the Supplementary Material.

### Statistical analysis

**Fig. 1:**
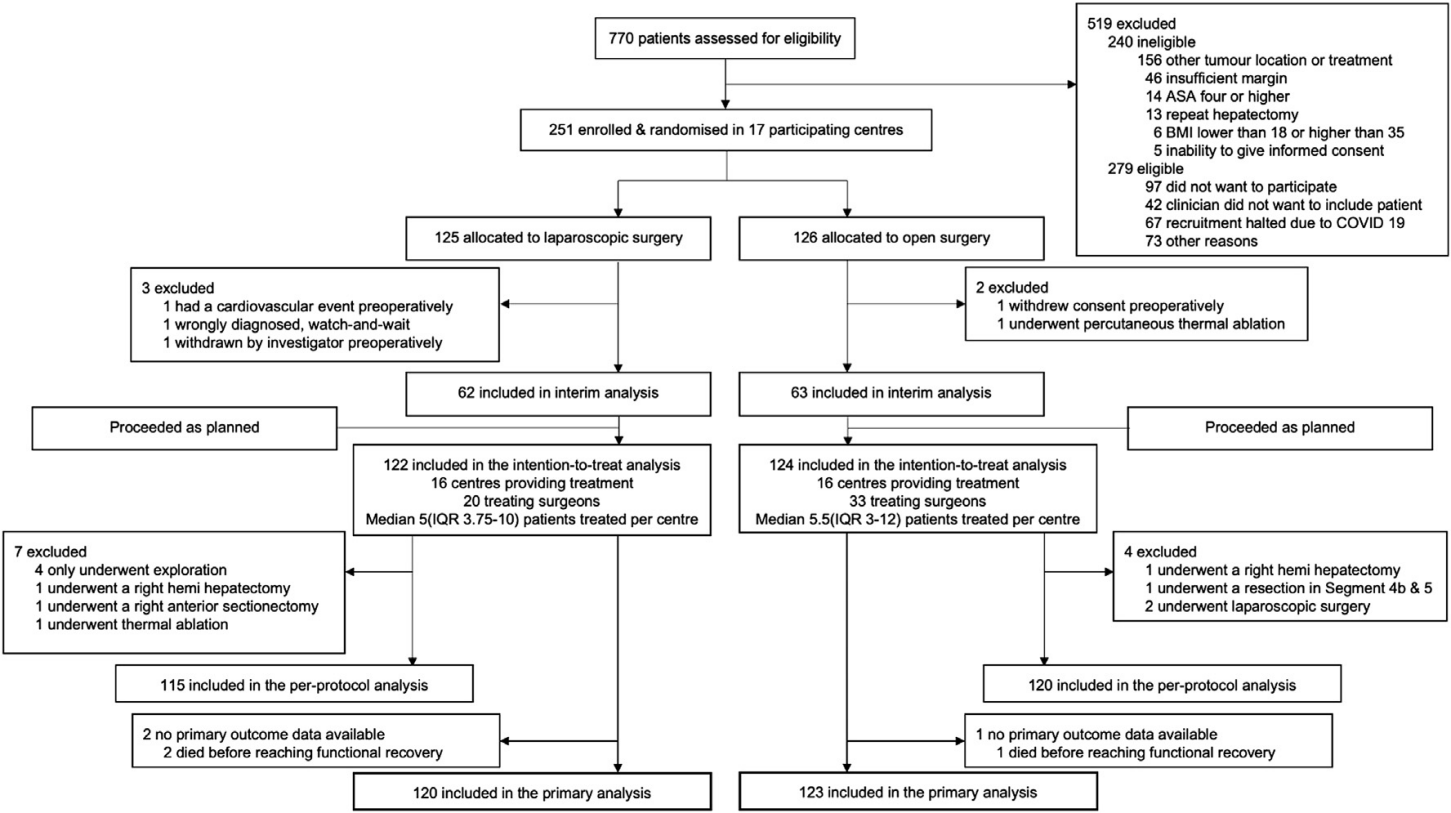
Trial profile.

All primary analyses were performed by intention-to-treat (ITT) with exclusion of patients who dropped out before receiving the allocated treatment (Fig. 1). In patients who were not functionally recovered at hospital discharge, the time to functional recovery was equal to the length of hospital stay (n = 2). An additional per-protocol analysis was performed including only patients who underwent the allocated procedure.

Categorical variables were reported as counts and percentages and compared using a Chi-square of Fisher's exact test. Continuous variables were reported as mean and standard deviation when normally distributed and as median and interquartile range (IQR) when not normally distributed and compare using an unpaired T-test or Mann–Whitney U test. The primary endpoint was measured in days and analysed using Poisson regression according to the distribution of the data, with treatment arm as the independent variable. The analysis was repeated after adding centre, lesion size smaller or equal/larger than 3 cm, age, sex and diagnosis (benign versus malignant) as covariates in the model. An additional analysis adjusted for allocation chance, to address potential baseline imbalance due to the minimisation procedure, was carried out. The effect estimates were converted, to obtain a relative difference compared to the reference group (open surgery). Subsequently, mixed-effects regression analyses were performed, with centre as a random effect to account for possible centre variation in the treatment effect. To be able to perform these analyses, centres that recruited five patients or less were pooled. Treatment by covariate interactions were explored.

The secondary endpoints were solely assessed using unadjusted and adjusted mixed effects analysis, using Poisson, Negative-binomial, or linear regression on log-transformed data, when appropriate, for continuous variables, logistic regression for binary variables, and Cox regression for time to the initiation of adjuvant chemotherapy. The secondary endpoint analyses were performed at a two-tailed alpha of 0.01, considering multiple outcome testing. Predefined exploratory subgroup analyses were performed. A post-hoc analysis was performed to estimate the intracluster correlation coefficients (ICC) of the participating centres for the primary and secondary endpoints. Data were analysed using R for Mac OS X version 4.2.1 (R Foundation for Statistical Computing, Vienna, Austria). Additional data on statistical methods, including details about the quality of life and health resource costs assessment, are available in the Supplementary Material.

### Role of the funding source

ORANGE Segments was an investigator-initiated study funded by Ethicon (Johnson & Johnson), Cancer Research United Kingdom (CRUK 12/048), the National Institute for Health and Care Research (NIHR) Southampton Biomedical Research Centre, and Maastricht University Medical Centre+. The funders had no role in the study design, data curation, data interpretation, manuscript writing or submission process. All authors were responsible for the design and conduct of this study, take full responsibility for the data curation and analysis, and have contributed to writing this report following the recommendations outlined in the CONsolidated Standards of Reporting Trials (CONSORT) statement.^19^

## Results

Between November 2017 and November 2021, 770 patients were screened for eligibility and 251 patients were randomly assigned to either laparoscopic (n = 125) or open (n = 126) resection of the posterosuperior liver segments. Five patients dropped out before surgery and therefore the ITT population included 246 patients (Fig. 1).

Functional recovery was achieved after a median of 3 days (IQR 3 to 5) in the patients allocated to laparoscopic surgery and 4 days (IQR 3 to 5) in the patients allocated to open surgery (Unadjusted difference −19.2%, 96% CI −28.8% to −8.4%, p < 0.001) (Table 3). This was paralleled by a median postoperative length of stay of 4 days (IQR 3 to 5) in the laparoscopic group and 5 days (IQR 4 to 7) in the open group (Unadjusted difference −22.7%, 99% CI −33% to −10.7%, p < 0.001).

**Table 3:** Primary and secondary endpoint analyses of the intention-to-treat population, stratified by the allocated surgical approach.

	Intention-to-treat population		Unadjusted difference (96% CI)^a^	p	Adjusted difference (96% CI)^b^	p
	(n = 246)					
	Laparoscopic	Open				
	(n = 122)	(n = 124)				
*Primary endpoint*						
Time to functional recovery, days	3 [3, 5]	4 [3, 5]	−19.2% (−28.8% to −8.4%)	<0.001	−21.5% (−30.9% to −10.8%)	<0.001
*Secondary endpoints*			Unadjusted difference (99% CI)^a^	p	Adjusted difference (99% CI)^b^	p
**Intraoperative**						
Operation duration, minutes	240 [186.3, 300]	200 [155, 270]	20.5% (8.1%–34.3%)	<0.001	20.4% (8.3%–33.8%)	<0.001
Estimated blood loss, milliliters	200 [100, 500]	250 [100, 400]	18.1% (−14.7% to 63.3%)	0.187	19.1% (−12.7% to 62.5%)	0.147
Unfavorable intraoperative incidents			OR 1.61 (0.56–4.65)	0.246	aOR 1.71 (0.56–5.17)	0.212
Satava 1	12 (9.9)	13 (10.5)				
Satava 2	6 (5)	0				
Satava 3	1 (0.8)	0				
Conversion						
To a hand-assisted procedure	1 (0.8)					
To an open procedure	16 (13.1)					
**Postoperative**						
Postoperative length of stay, days	4 [3, 5]	5 [4, 7]	−22.7% (−33% to −10.7%)	<0.001	−23.1% (−33.3% to −11.2%)	<0.001
90-day overall morbidity	17 (14.2)	28 (23.3)	OR 0.47 (0.18–1.22)	0.040	aOR 0.44 (0.17–1.18)	0.032
Mild (Clavien-Dindo grade I or II)	11 (9.2)	17 (14.2)	OR 0.48 (0.14–1.65)	0.125	aOR 0.44 (0.12–1.54)	0.089
Severe (Clavien-Dindo grade ≥ IIIA)	6 (5)	11 (9.2)	OR 0.52 (0.13–2.05)	0.218	aOR 0.51 (0.12–2.11)	0.219
Liver-specific	7 (5.8)	14 (11.7)	OR 0.47 (0.12–1.56)	0.118	aOR 0.45 (0.12–1.68)	0.119
90-day readmission	7 (5.9)	12 (10.1)	OR 0.54 (0.13–1.94)	0.227	aOR 0.50 (0.12–2.02)	0.201
90-day or in-hospital mortality	5 (4.2)	1 (0.8)	OR 5.17 (0.30–89.01)	0.137	aOR 5.11 (0.29–90.15)	0.143
Complication-related	3 (2.5)	1 (0.8)	OR 3.06 (0.15–61.4)	0.336	aOR 2.92 (0.14–60.98)	0.363
Disease-related	2 (1.7)	0				
Comprehensive Complication Index			OR 0.52 (0.19–1.42)	0.094	aOR 0.48 (0.17–1.39)	0.076
Category A (Score 0–20)	108 (90)	99 (82.5)				
Category B (Score 20–60)	9 (7.5)	20 (16.7)				
Category C (Score 60–100)	3 (2.5)	1 (0.8)				
Delay of discharge after functional recovery	39 (33.6)	55 (45.1)	OR 0.52 (0.23–1.17)	0.037	aOR 0.49 (0.21–1.12)	0.026
Resection margin^c^			OR 0.77 (0.25–2.35)	0.539	aOR 0.81 (0.26–2.52)	0.625
R0: resection margin ≥1 mm	93 (87.7)	97 (85.8)				
R1: resection margin <1 mm	12 (11.3)	15 (13.3)				
R2: macroscopically irradical	1 (0.9)	1 (0.9)				
Time to initiation of adjuvant chemotherapy, days^d^	44 [35, 60.8]	61 [45.8, 72.8]	HR 1.52 (0.67–3.43)	0.190	aHR 1.26 (0.53–3.01)	0.500
Incisional hernia at one year follow-up	6 (9.2)	5 (6)	OR 1.54 (0.21–11.5)	0.578	aOR 0.87 (0.08–8.95)	0.875

Primary outcome data was available for all patients, except those that deceased before reaching the primary endpoint (two in the laparoscopic group and one in the open group).

Values are expressed in counts (percentages) or in median [IQR]. Abbreviations: CI, confidence interval; OR, odds ratio; aOR, adjusted odds ratio.

a The open group was consistently used as reference group.

b Adjusted for sex, age, benign/malignant lesion type, lesion size, and centre.

c In case of malignancy.

d When treated with adjuvant chemotherapy, patients that underwent a subsequent resection of the primary and received chemotherapy after this procedure were excluded.

Patient characteristics at baseline were well balanced between the groups. The majority of patients were male (n = 169, 68.7%) and had a performance status of 0 (n = 188, 76.4%). Nearly all patients had a preoperative diagnosis of cancer (n = 225, 91.5%), with half being colorectal liver metastasis (n = 127, 51.6%) (Table 1). Nearly one third of the patients were preoperatively treated with systemic chemotherapy (n = 70, 28.5%) for a median of six cycles, most frequently with FOLFOX (Table 1 & Supplementary Table S1). Data on ethnicity were not collected.

**Table 1:** Baseline characteristics of the intention-to-treat population, stratified by the allocated surgical approach.

Baseline characteristics	Intention-to-treat population	
	(n = 246)	
	Laparoscopic	Open
	(n = 122)	(n = 124)
Sex		
Male	81 (66.4)	88 (71)
Female	41 (33.6)	36 (29)
Age, years	68 [60.3, 74]	68 [58, 75]
BMI, kilogram divided by square meter	26.3 [23.5, 28.7]	26.7 [24.2, 29.1]
ASA classification		
I: healthy	12 (9.8)	8 (6.5)
II: mild systemic disease	77 (63.1)	77 (62.1)
III: severe systemic disease	33 (27.0)	39 (31.5)
ECOG-performance status score		
O: asymptomatic, normal activity	93 (76.2)	95 (76.6)
1: symptomatic, normal activity	27 (22.1)	25 (20.2)
2: symptomatic, <50% bedridden	2 (1.6)	4 (3.2)
Comorbidity	88 (72.1)	93 (75)
Cardiovascular	72 (59)	79 (63.7)
Respiratory	16 (13.1)	28 (22.6)
Other	6 (4.9)	8 (6.5)
Previous abdominal surgery	78 (63.9)	80 (64.5)
Preoperative systemic treatment with chemotherapy	37 (30.3)	33 (26.6)
Preoperative diagnosis		
Benign	10 (8.2)	4 (3.2)
Malignant		
Colorectal metastasis	63 (51.6)	64 (51.6)
Non-colorectal metastasis	7 (5.7)	7 (5.6)
Hepatocellular carcinoma	33 (27)	39 (31.5)
Cholangiocarcinoma	5 (4.1)	4 (3.2)
Other	1 (0.8)	2 (1.6)
Unknown	3 (2.5)	4 (3.2)
Preoperative size of the largest lesion		
<3 cm	64 (52.5)	66 (53.2)
≥3 cm	58 (47.5)	58 (46.8)
Extrahepatic metastases	11 (9)	6 (4.8)

Values are expressed in counts (percentages) or in median [IQR].

There were no statistically significant differences between the groups.

Abbreviations: BMI, body mass index; ASA, American Society of Anesthesiologists; ECOG, Eastern Cooperative Oncology Group.

The surgical characteristics are reported in Table 2. There were no statistically significant differences between the treatment groups, although patients in the laparoscopic group more often underwent resections in segment 7 (46.2% versus 30.6%), while patients in the open group more often underwent resections in segment 8 (49.6% versus 30.8%). Additionally, patients in the open group more often underwent concurrent extrahepatic procedures (29% versus 18.9%). A large proportion of the patients underwent a resection of a single lesion, namely 85.8% in the laparoscopic group and 80.8% in the open group.

**Table 2:** Surgical characteristics of the intention-to-treat population, stratified by the allocated surgical approach.

Surgical characteristics	Intention-to-treat population	
	(n = 246)	
	Laparoscopic	Open
	(n = 122)	(n = 124)
Number of lesion(s)		
Single	103 (85.8)	97 (80.8)
Multiple	17 (14.2)	23 (19.2)
Location of tumor and type of resection^a^		
Segment 4A	14 (11.7)	14 (11.3)
Anatomical	6 (46.2)	3 (21.4)
Non-anatomical	7 (53.8)	11 (78.6)
Segment 6/7	25 (21)	23 (18.5)
Anatomical	14 (58.3)	11 (47.8)
Non-anatomical	10 (41.7)	12 (52.2)
Segment 7	55 (46.2)	38 (30.6)
Anatomical	19 (35.2)	13 (34.2)
Non-anatomical	35 (64.8)	25 (65.8)
Segment 8	37 (30.6)	62 (50)
Anatomical	9 (24.3)	21 (33.9)
Non-anatomical	28 (75.7)	41 (66.1)
Other location	0	1 (0.8)
Additional local treatment		
Resection	4 (3.3)	7 (5.6)
Ablation	3 (2.5)	2 (1.6)
Concurrent extrahepatic procedures	23 (18.9)	36 (29)
Cholecystectomy	20 (87)	31 (86.1)
Cholecystectomy, lymphadenectomy	1 (4.3)	1 (2.8)
Cholecystectomy, open inguinal hernia repair	1 (4.3)	0
Lymphadenectomy	1 (4.3)	1 (2.8)
Incisional hernia repair	0	1 (2.8)
Mastectomy, axillary lymph node clearance	0	1 (2.8)
Para duodenal lymph node biopsy	0	1 (2.8)

Values are expressed in counts (percentages).

There were no statistically significant differences between the groups.

a Counts exceed the sample because some patients had multiple tumors, type of resection only specified when performed.

The operative time was longer in patients allocated to laparoscopic surgery, with a median of 240 minutes (IQR 186.3–300), compared to 200 minutes (IQR 155–270) in patients allocated to open surgery (Unadjusted difference 20.5%, 99% CI 8.1%–34.3%, p < 0.001) (Table 3). Blood loss and unfavourable incidents were similar between the groups. In the laparoscopic group, 17 procedures (13.9%) were converted to an open (n = 16) or hand-assisted (n = 1) approach. Of these 17 converted procedures, 5 conversions (29.4%) were performed in an emergency setting (reactive) due to bleeding or intolerance of the pneumoperitoneum, while the remaining 12 conversions (70.6%) were proactive (non-emergent) due to encountered technical difficulties such as dense adhesions or oncological reasons (Supplementary Table S2).

At 90 days postoperatively, trends for overall morbidity, severe morbidity, liver-specific morbidity, and readmission favoured the laparoscopic group but none of these met statistical significance (Table 3). The most commonly encountered adverse events were surgical site infections and pulmonary complications (Table 4). Five patients allocated to laparoscopic surgery (4.2%) and one patient allocated to open surgery (0.8%) died within 90 days postoperatively (OR 5.17, 99% CI 0.30–89.01, p = 0.137). Four deaths (laparoscopic n = 3, open n = 1) were related to complications of surgery (Supplementary Table S4). A further two deaths in the laparoscopic group were as a result of cancer progression. In these patients the procedure was aborted intraoperatively as disease progression was noted during diagnostic laparoscopy (Supplementary Table S5).

**Table 4:** Comprehensive overview of adverse events.

Adverse event	Intention-to-treat population		RR (95% CI)	RD (95% CI)
	(n = 246)			
	Laparoscopic	Open		
	(n = 122)	(n = 124)		
**Total number of AE**	17 (13.9)	28 (22.5)	0.61 (0.35–1.06)	−0.08 (−0.18 to 0.00)
Surgical Site infection	4 (3.2)	7 (5.6)	0.58 (0.17–1.93)	−0.02 (−0.07 to 0.02)
Superficial	1 (0.8)	2 (1.6)	0.50 (0.04–5.53)	0.00 (−0.03 to 0.01)
Deep	0	0	1.01 (0.02–50.81)	0.00 (0.00–0.00)
Organ space	3 (2.5)	5 (4.0)	0.60 (0.14–2.49)	−0.01 (−0.05 to 0.02)
Posthepatectomy bile leak	0	5 (4.3)	0.09 (0.00–1.65)	−0.04 (−0.07 to −0.00)
Posthepatectomy hemorrhage	0	1 (0.8)	0.33 (0.01–8.23)	−0.00 (−0.02 to 0.00)
Posthepatectomy liver failure	1 (0.8)	0	3.04 (0.12–74.12)	0.00 (−0.00 to 0.02)
Pulmonary complication	4 (3.2)	4 (3.2)	1.01 (0.26–3.97)	0.00 (−0.04 to 0.04)
Kidney failure	1 (0.8)	0	3.04 (0.12–74.12)	0.00 (−0.00 to 0.02)
Delirium	0	1 (0.8)	0.33 (0.01–8.23)	−0.00 (−0.02 to 0.00)
**AEs of Special Interest**				
Air embolism	0	0	1.01 (0.02–50.81)	0.00 (0.00–0.00)
**Interventions**	3 (2.5)	10 (8.0)	0.30 (0.08–1.08)	−0.05 (−0.11 to −0.00)
Reoperation	0	1 (0.8)	0.33 (0.01–8.23)	−0.00 (−0.02 to 0.00)
Endoscopic intervention	0	3 (2.5)	0.14 (0.00–2.78)	−0.02 (−0.05 to 0.00)
Percutaneous intervention	3 (2.5)	6 (4.8)	0.50 (0.13–1.98)	−0.02 (−0.07 to 0.02)
**90-day mortality**	5 (4.1)	1 (0.8)	5.05 (0.60–42.87)	0.03 (−0.00 to 0.07)
Complication-related	3 (2.5)	1 (0.8)	3.04 (0.32–28.91)	0.01 (−0.01 to 0.04)
Oncology related	2 (1.6)	0	5.08 (0.24–104.76)	0.01 (−0.00 to 0.03)

Abbreviations: AE, adverse event; RR, relative risk; RD, risk difference.

**Fig. 2:**
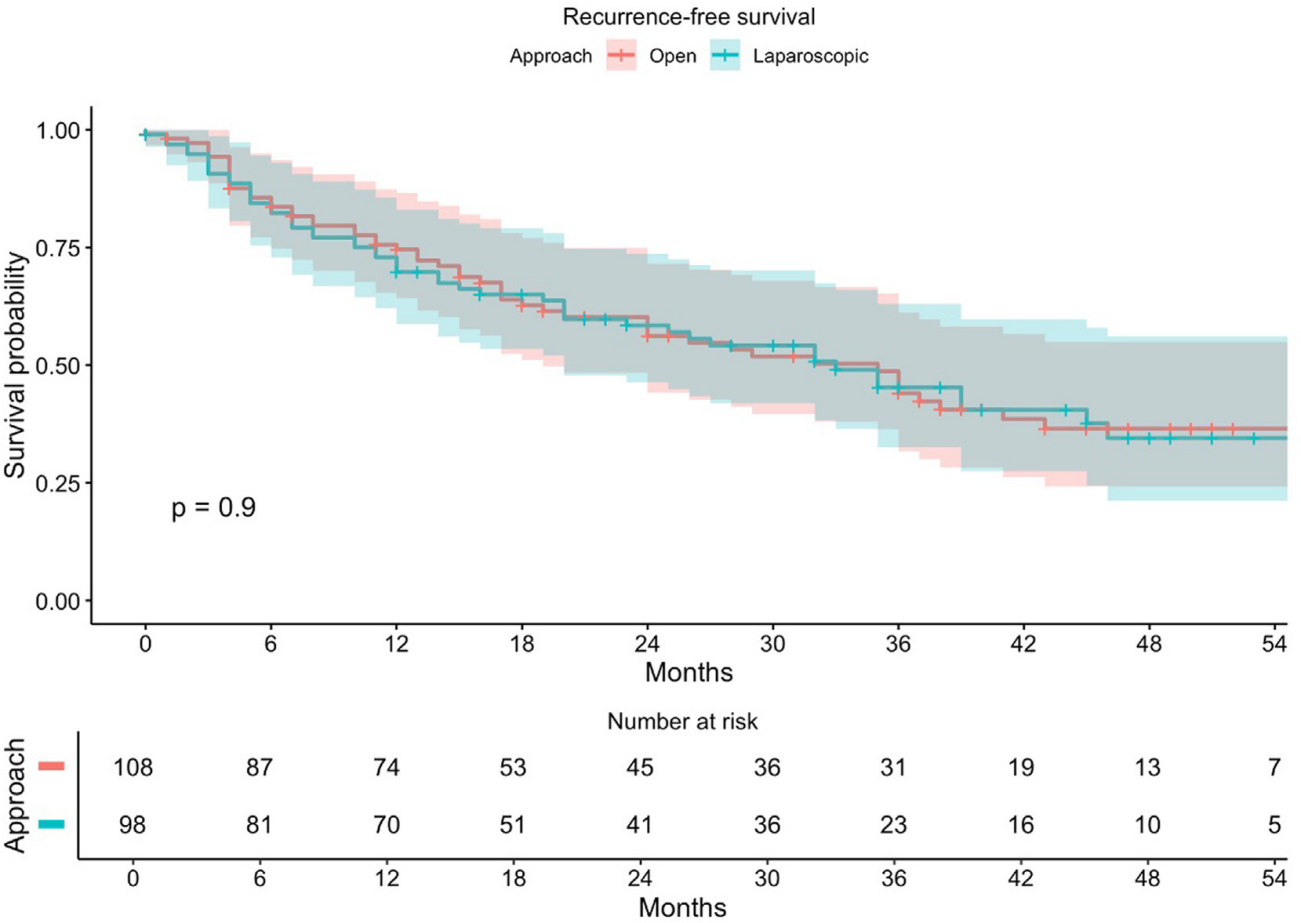
Recurrence-free survival of the patients in the intention-to-treat population that underwent surgery for malignancies.

**Fig. 3:**
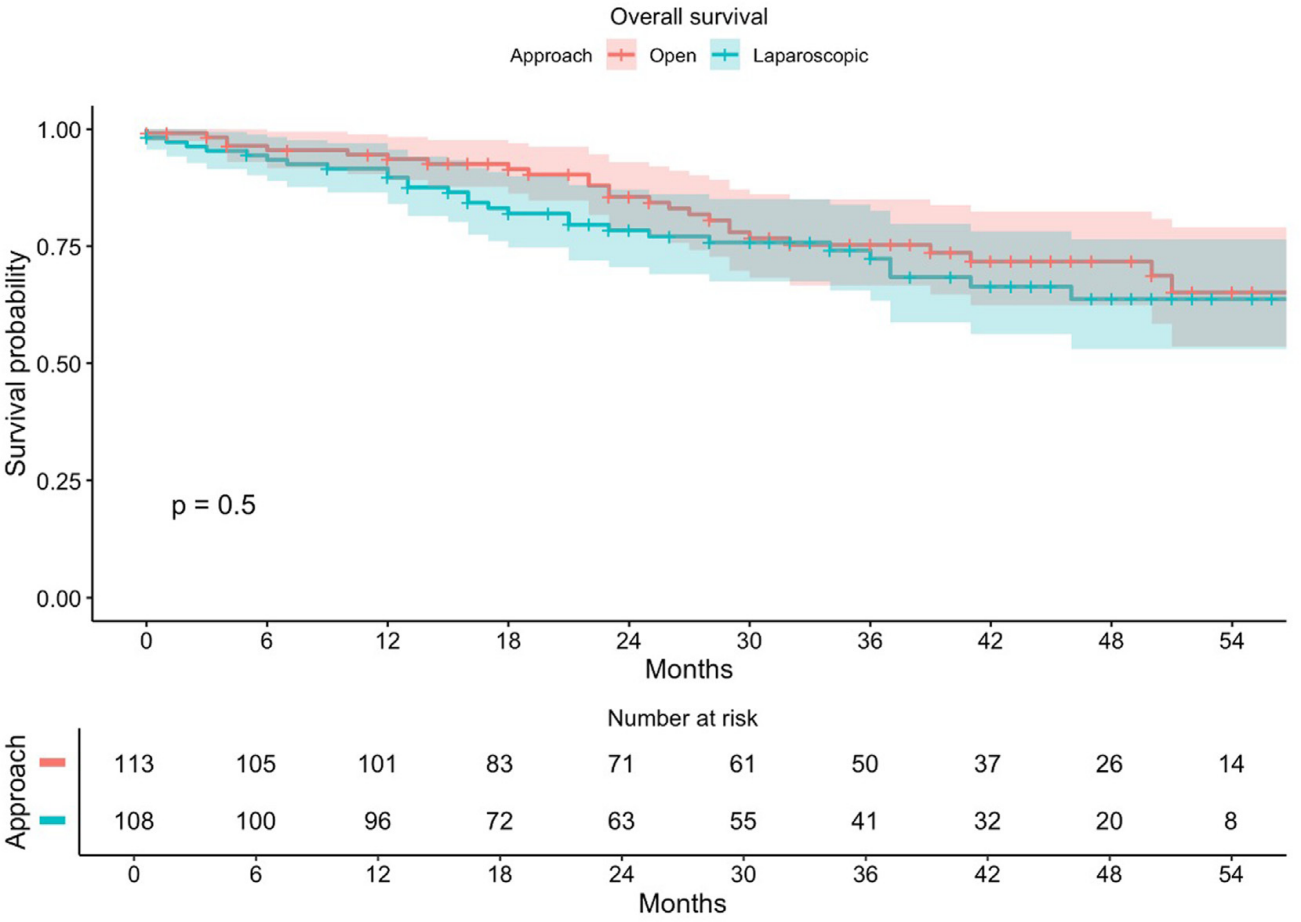
Overall survival of the patients in the intention-to-treat population that underwent surgery for malignancies.

In the patients that had a histopathological diagnosis of malignant disease (n = 224), the radical resection margin rate was 87.7% in the laparoscopic group and 85.8% in the open group (p = 0.539) (Table 3). The median time to initiation of adjuvant chemotherapy was 44 days (IQR 35–60.8) in the laparoscopic group and 61 days (IQR 45.8–72.8) in the open group (HR 1.52, 99% CI 0.67–3.43, p = 0.190). After a median follow-up period of 37 (IQR 21–50) months, 115 (51.3%) of the 224 patients had a recurrence. In the laparoscopic group, 57 (54.3%) of the 111 patients were affected by a recurrence, and in the open group 58 (51.3%) of the 113 patients (p = 0.900) (Supplementary Table S5 and Fig. 2). At a median follow-up of 38 (IQR 21–50) months 56 patients died (25%), 29 (26.9%) of the 111 patients in the laparoscopic and 27 (23.9%) of the 113 patients in the open group, respectively (p = 0.500) (Supplementary Table S5 and Fig. 3). At 12 months follow-up, an incisional hernia had been diagnosed in six patients (9.2%) in the laparoscopic group and five patients (6%) in the open group (OR 1.54, 99% CI 0.21–11.5, p = 0.578).

**Supplementary Figure S1:**
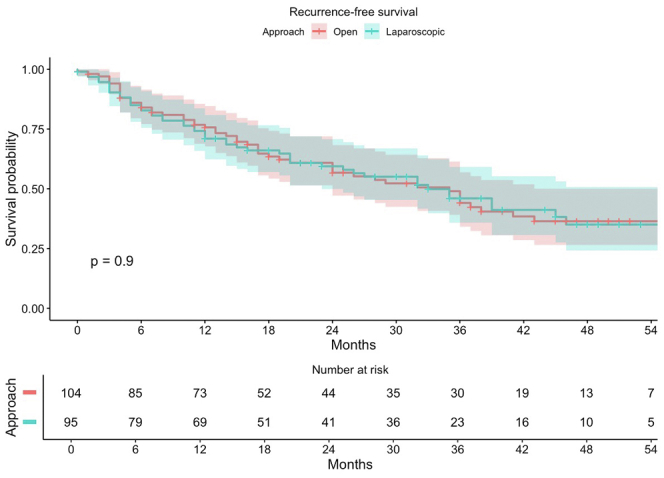
Recurrence-free survival of the patients in the per-protocol population that underwent surgery for malignancies.

**Supplementary Figure S2:**
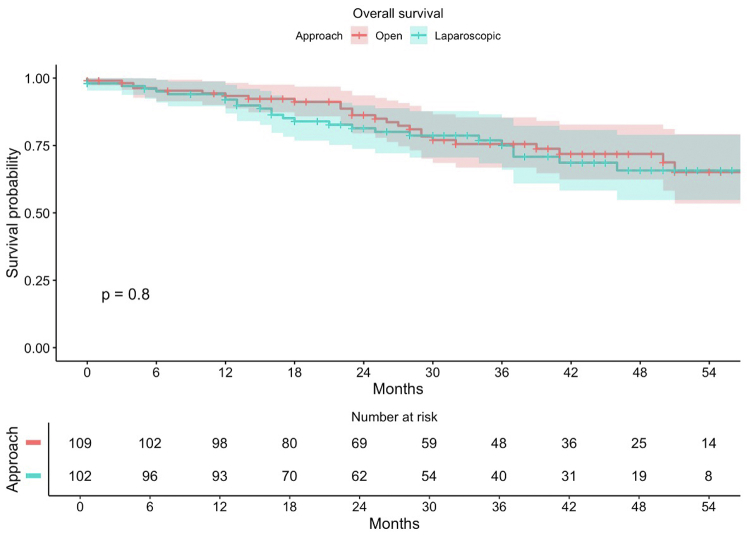
Overall survival of the patients in the per-protocol population that underwent surgery for malignancies.

**Supplementary Figure S3:**
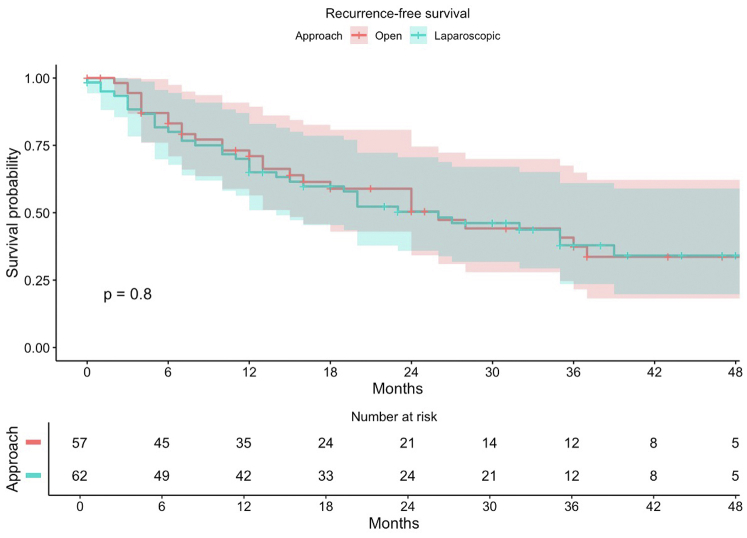
Recurrence-free survival of the patients in the intention-to-treat population that underwent surgery for colorectal liver metastases.

**Supplementary Figure S4:**
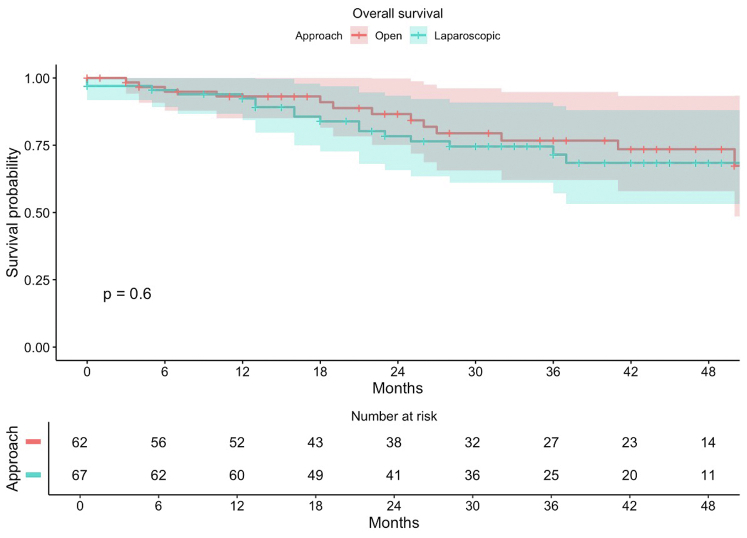
Overall survival of the patients in the intention-to-treat population that underwent surgery for colorectal liver metastases.

**Supplementary Figure S5:**
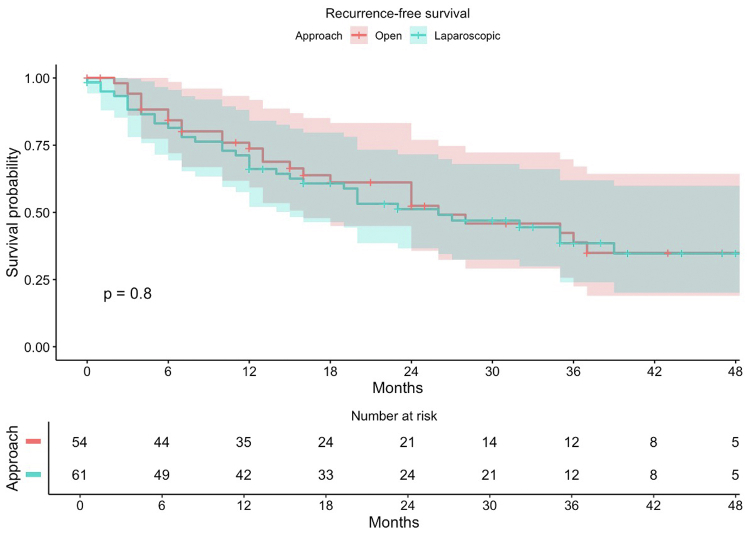
Recurrence-free survival of the patients in the per-protocol population that underwent surgery for colorectal liver metastases.

**Supplementary Figure S6:**
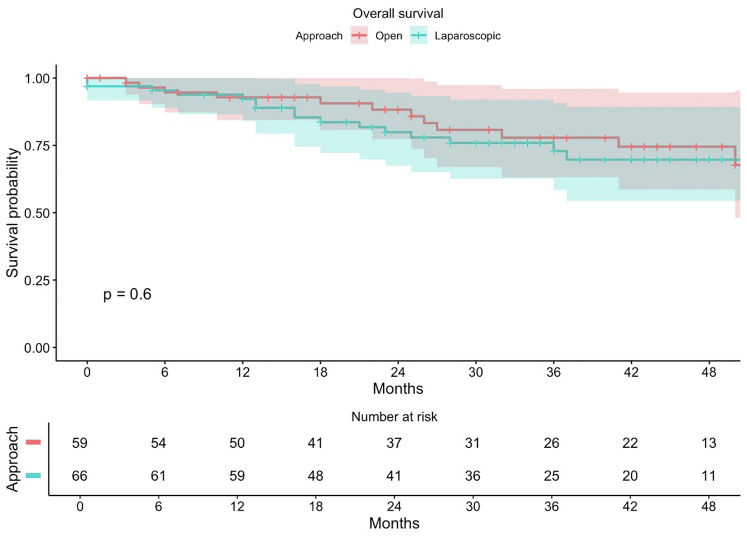
Overall survival of the patients in the per-protocol population that underwent surgery for colorectal liver metastases.

In the included patients who underwent surgery for colorectal liver metastases (n = 129), the tumour-free resection margin rate was 85.1% in the laparoscopic group and 82% in the open group (p = 0.313) (Supplementary Table S6). After a median follow-up period of 36 (IQR 21–47) months, 73 (56.6%) of these 129 patients had a recurrence. In the laparoscopic group, 40 (59.7%) of the 67 patients were affected by a recurrence, and in the open group 33 (53.2%) of the 62 patients (p = 0.800) (Supplementary Table S6 & Supplementary Figure S3). Of these patients with colorectal liver metastases, 31 (24%) died at a median follow-up of 38 (IQR 23–49) months, 17 (25.4%) of the 67 patients in the laparoscopic and 14 (22.6%) of the 62 patients in the open group, respectively (p = 0.600) (Supplementary Figure S4). The per-protocol analyses were consistently in line with the results of the ITT analysis (Supplementary Tables S7–S11, Supplementary Figures S1, S2, S5 and S6).

**Fig. 4:**
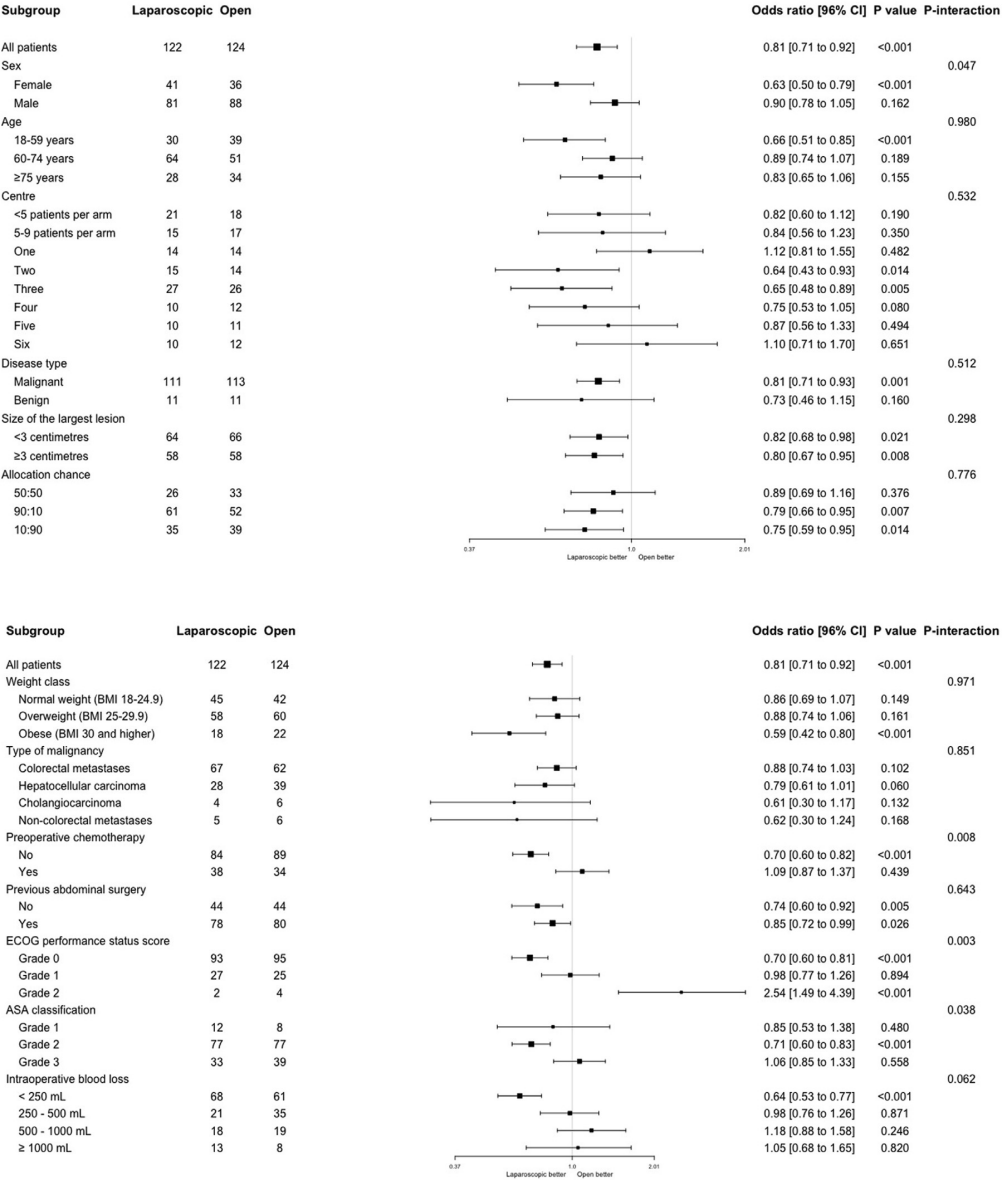
Exploratory subgroup analyses of primary endpoint.

The prespecified subgroup analyses of the primary endpoint demonstrated statistically significant subgroup effects according to patient's sex, the presence or absence of preoperative chemotherapeutical treatment and ASA grade (Fig. 4). Laparoscopic surgery was associated with a greater treatment effect in patients of the female sex, patients that were not treated with preoperative chemotherapy and patients with an ASA grade of 2. In patients with an ECOG score of 2, the open approach was associated with a shorter time to functional recovery (OR 2.54, 96% CI 1.49–4.39, p < 0.001), although only 6 patients were included in this subgroup (Fig. 4). Data regarding blinding efficacy was available for 58.3% of patients, 19 of 70 patients (27.1%) that underwent laparoscopic surgery and 25 of 67 patients (35.7%) that underwent open surgery reported to have undergone the opposite procedure.

**Supplementary Figure S7:**
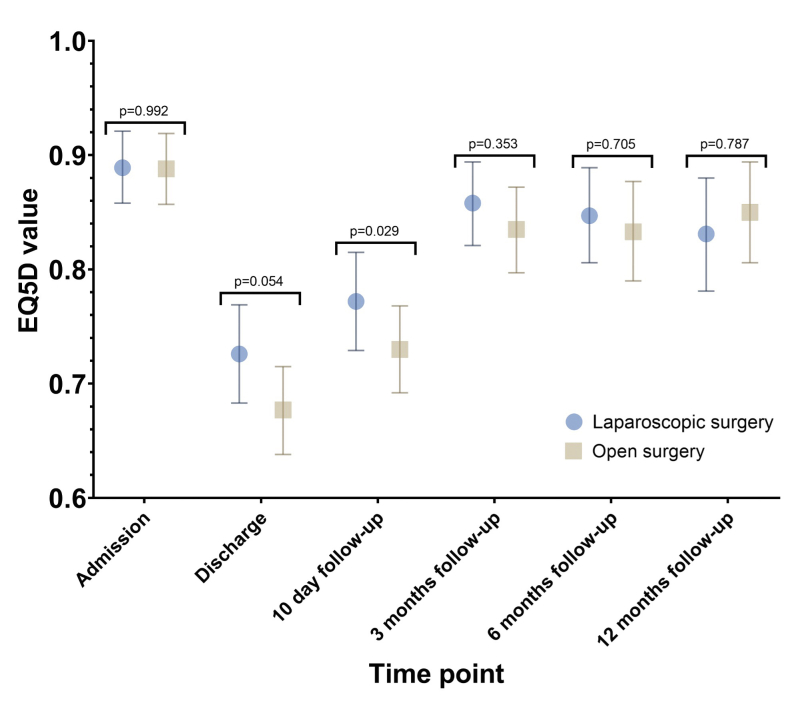
EQ5D values over time for the dimensions mobility, self-care, usual activities, pain/discomfort and anxiety/depression.

**Supplementary Figure S8:**
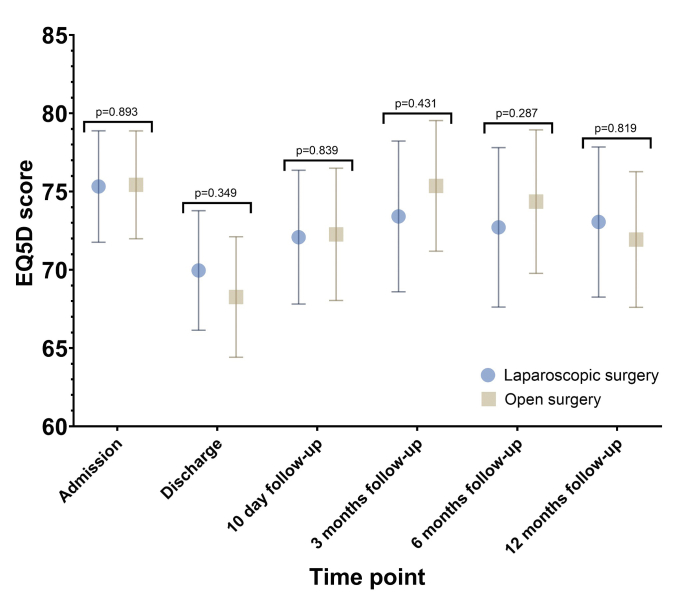
EQ5D visual analogue scale quality of life scores over time.

**Supplementary Figure S9:**
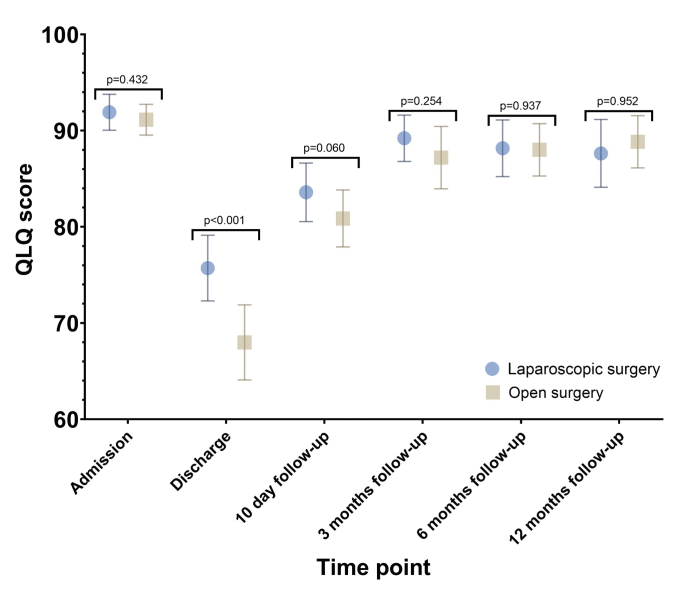
QLQ-C30 summary scores over time.

**Supplementary Figure S10:**
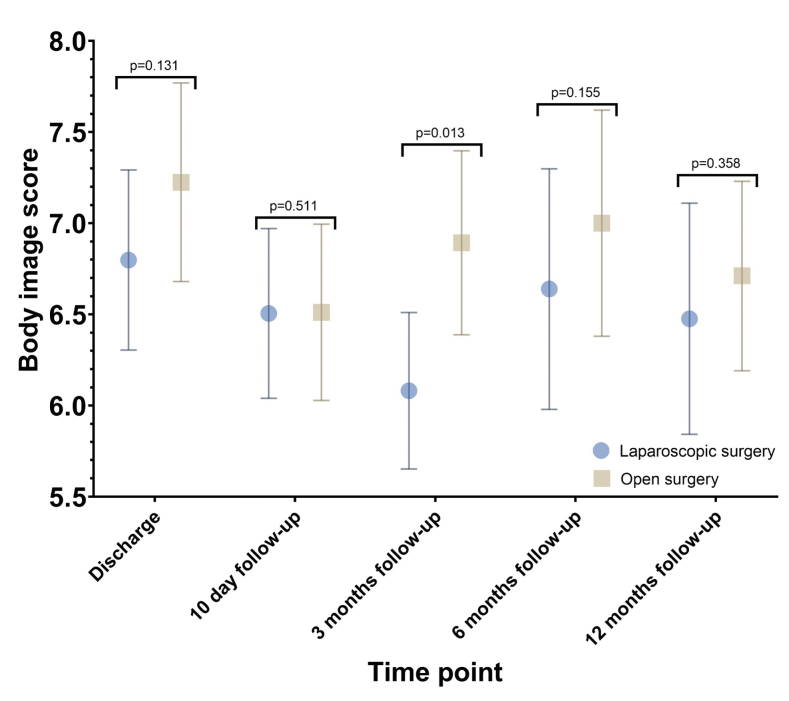
Body image scores over time.

**Supplementary Figure S11:**
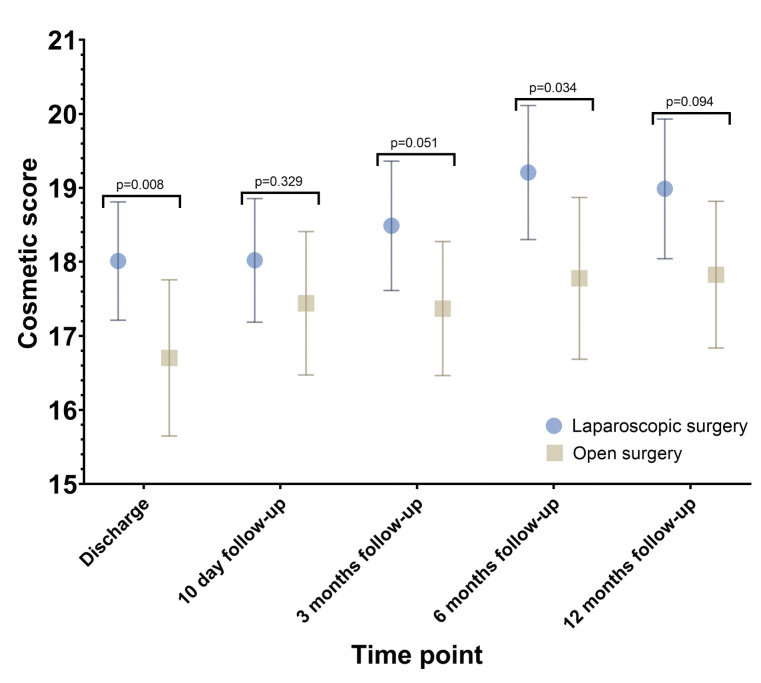
Cosmetic scores over time.

In terms of quality of life, laparoscopic surgery was associated with numerically higher EQ5D health state description values until 6 months follow-up, but these differences did not reach statistical significance (Supplementary Figure S7). There were also no statistically significant differences between the two groups in EQ5D visual analogue scale scores and QLQ-C30 summary scores, besides superior QLQ-C30 summary scores in the laparoscopic group at discharge (Mean score 75.7 versus 68, respectively, p < 0.001) (Supplementary Figures S8 & S9). Patients allocated to laparoscopic surgery reported numerically better body image and cosmetic scores up till 12 months postoperatively, but these differences also did not reach statistical significance (Supplementary Figures S10 & S11). The mean health resource costs were €11.249 in the laparoscopic group (99% BCaCI €10.109–€13.205) and €11.848 (99% BCaCI €10.852–€13.308) in the open group (Mean difference €598, 99% BCaCI −€1377 to €2364, p = 0.408).

## Discussion

This first randomised trial to assess laparoscopic versus open parenchyma preserving resection in the posterosuperior segments of the liver demonstrated a one-day reduction in time to functional recovery and similar reduction of length of stay. Nevertheless, the hypothesized difference in time to functional recovery of two days was not reached.

These results are in line with the recently published ORANGE II PLUS trial which similarly demonstrated a reduction in time to functional recovery by one day, from six days to five days, with the laparoscopic approach to hemi hepatectomy.^20^ The time to functional recovery is appropriately shorter in both groups of the ORANGE Segments trial compared with the ORANGE II PLUS trial since the liver resection is less extensive. The hypothesized difference in time to functional recovery of two days was not met, likely due to the extensive experience of the participating centres and the implemented enhanced recovery after surgery programs. However, we postulate that the observed reduction in time to functional recovery of nearly 20% is a meaningful difference for patients.

In accordance with the results reported in the ORANGE II PLUS trial there was no evidence that oncological outcomes were compromised. The R0 resection rates were excellent in both groups and higher than those observed in the subgroup of patients with lesions in the posterosuperior segments in the OSLO-COMET single centre randomised controlled trial.^21^ This may in part reflect improvements in imaging and surgical technique, but more likely relates to selection since the OSLO-COMET trial included patients with a history of liver surgery and a larger proportion of the patients were affected by multiple liver lesions. Sites of recurrence in the ORANGE Segments trial were similarly reassuring with no apparent differences. The ability to compare longer term outcomes such as recurrence-free and overall survival is limited by the patient numbers, length of follow-up and the fact that disease characteristics (e.g., tumour type and presence or absence of extrahepatic disease) were not a stratification factor for randomisation but certainly no differences were evident between the open and laparoscopic groups. Notably, it has been suggested that patients might benefit from laparoscopic surgery when a subsequent surgery is needed (e.g., in case of a disease recurrence that can be treated with repeat resection or transplantation), as laparoscopic surgery is associated with less severe adhesion formation.^22^^,^^23^

In contrast to the ORANGE II PLUS trial, the ORANGE Segments trial did not demonstrate a statistically significant reduction in time to the initiation of adjuvant chemotherapy. In predefined subgroup analyses, which should be cautiously interpreted and are exploratory in nature, the laparoscopic approach had a greater treatment effect in the absence of preoperative chemotherapy and a high ASA grade. The time to functional recovery achieved across both the laparoscopic and open groups of the trial, just three and four days respectively, were impressive. As such it is postulated that the relatively small incremental gain from the laparoscopic approach, of just one day in recovery, might only be possible in patients with sufficient physiological reserve.

In terms of safety, no statistically significant differences were found for 90-day morbidity and mortality. It is important to note the 90-day mortality rate which was relatively high for minor liver resections with five deaths in the laparoscopic group and one death in the open group. Although two of the five patients in the laparoscopic group died due to progressive disease an incidence of three deaths is higher than expected for a resection of this level of complexity performed in experienced centres. This difference between the two groups of the trial does not reach statistical significance and indeed the trial was not powered to detect such a difference. The laparoscopic approach was associated with at least comparable patient reported outcomes, in terms of quality of life, body image and cosmesis, at similar health resource costs. These findings are in line with the results of earlier randomised trials comparing minimally invasive and open surgery, and are important in the era of patient centred care.^20^^,^^24^, ^25^, ^26^

This study demonstrates that the laparoscopic approach can be considered a valid alternative for these complex resections in centres that have gained sufficient experience in laparoscopic hemi hepatectomy and resections in the anterolateral segments of the liver. Although the gain here is incremental these data show tangible patient benefit provided the technique can be introduced without increasing the incidence of perioperative complications. Hence, in introducing these procedures surgeons must seek appropriate support from expert centres including both mentorship and proctorship.^27^^,^^28^

Future randomised studies should investigate if the robotic approach offers any benefits over the laparoscopic approach when performing specific liver surgical procedures, as several retrospective studies have pointed in this direction.^29^^,^^30^ These studies should not only focus on key perioperative and oncological outcomes, but also on surgeon ergonomics, environmental footprint, patient-reported outcomes and cost-effectiveness.

The results of this study should be interpreted in light of several limitations. First, there were centre-specific perioperative care protocols but there was no uniform protocol for the trial. It was decided not to implement such a protocol due to the pragmatic character of the trial and the doubtful feasibility of changing daily clinical practice in every participating centre. Importantly, centre effects would likely influence the outcomes of both laparoscopic and open procedures, and the treatment effect remained present when correcting for them. Second, a potential influence of the learning curve cannot be excluded. While all liver surgeons participating in this trial had substantial experience in performing both laparoscopic and open liver resections, the learning curve is a continuum, and with further experience a greater treatment effect might be observed. Third, the operating theatre staff was not blinded, and any contamination between operating theatre staff and outcome assessors possibly introducing bias cannot be ruled out. However, within centres patients followed the same perioperative care pathway, irrespective of the allocated approach. Additionally, the criteria a patient needs to meet to be deemed functionally recovered are relatively objective, decreasing the potential for inter-rater variability because of subjectivity. Therefore, it seems unlikely that this has greatly influenced the observed results. Fourth, the sample size was relatively small, increasing the risk of type 2 errors. This should mainly be kept in mind while interpreting the secondary endpoint data. Fifth, data on ethnicity was not collected, therefore it is unclear whether a diverse patient population was included. It is important to note that the results of this trial should not be extrapolated to patients with extensive disease or those that have a history of previous hepatic surgery due to its eligibility criteria. Furthermore, the results are not generalisable to all surgical centres due to the fact that this trial was conducted in specialist centres. The strengths of this trial are the patient-blinding, the pragmatic multicentre design, standardized perioperative care in the setting of ERAS, and the high retention rate for early outcomes.

In conclusion the ORANGE Segments trial demonstrated a one-day reduction (from four to three days) in time to functional recovery after laparoscopic parenchymal preserving resections in the posterosuperior liver segments, as compared to the open approach. However, this reduction in time to functional recovery did not meet the hypothesized difference in time to functional recovery of two days.

## Contributors

Study conceptualisation and methodology: Fichtinger, van Dam, Primrose, Abu Hilal.

Acquisition of data: All authors.

Formal analysis and interpretation of data: Sijberden, Kuemmerli, Brandts, Ferrari, Pugh, Primrose, Abu Hilal.

Writing–original draft: Sijberden, Kuemmerli, Brandts, Ferrari, Pugh, Primrose, Abu Hilal.

Writing–review and editing: All authors.

## Data sharing statement

Data supporting this work and additional related documents (i.e., informed consent forms) are available for qualified researchers upon reasonable request from the corresponding author. Data will include individual participant data that underlie the results reported in this article.

## Declaration of interests

The authors declare that they have no known competing financial interests or personal relationships that could have appeared to influence the work reported in this paper. However, outside of the submitted work Ronald van Dam received grants from Abbott Laboratories and Guerbet for investigator-initiated studies, consulting fees from BARCO and receipt of materials from Abbott laboratories. Siân Pugh reported speakers honoraria from Merck, Takeda and Servier and support for attending meetings and/or travel from Takeda.

## Appendix A Supplementary data

Supplementary Tables

Orange Segments

1 - DSMB charter

Updated version Statistical Analysis Plan -ORANGE trial-1
