# Supplementary material for: Laparoscopic versus open parenchymal preserving liver resections in the posterosuperior segments (ORANGE Segments): a multicentre, single-blind, randomised controlled trial
Source: Lancet Reg Health Eur. 2025 Feb 20;51:101228. doi: 10.1016/j.lanepe.2025.101228 (PMC11889631; doi:10.1016/j.lanepe.2025.101228)
Supplement: Supplementary Tables [file mmc1.docx]

**SUPPLEMENTARY MATERIAL**

**Outcome definitions**

Intraoperative unfavourable incidents were defined and graded according to the Satava classification, adapted for liver surgery.^1^ Postoperative morbidity was defined and graded according to Clavien-Dindo and reported as mild (Clavien-Dindo grade I or II) or severe (Clavien-Dindo grade ≥ IIIa).^2^ Furthermore, the Comprehensive Classification Index (CCI) was used in order to assess all postoperative complications.^3^ Liver-specific morbidity comprised operative mortality, ascites, postoperative liver failure, bile leakage, intra-abdominal haemorrhage or abscess.^4^

**Quality of life and health resource costs**

Quality of life outcomes were analysed using linear mixed models. The participating centre (as random effect) and allocated surgical approach (i.e., laparoscopic or open) were included in these models. Mean differences in quality of life per time point were determined. A comparisons of health resource costs was performed, incorporating surgical treatment (theatre, anaesthesia-related and personnel costs), inpatient stay (general ward and intensive care unit, including readmissions) and complications (i.e., transfusions and endoscopic, radiological or surgical reinterventions) as major components. Costs are expressed in Euros (€) and represent the Dutch setting, reference year 2022. The costs of laparoscopic and open surgery were compared using nonparametric bootstrapping (1000 samples) and were reported as mean differences with their respective two-sided 95% confidence interval, calculated using the bias-corrected and accelerated bootstrap (BCaCI). A comprehensive quality of life and health economic evaluation will be performed as a sub study and will include outpatient costs, micro-costs and quality adjusted life years.

**References**

**1**. Kazaryan AM, Røsok BI, Edwin B: Morbidity Assessment in Surgery: Refinement Proposal Based on a Concept of Perioperative Adverse Events. ISRN Surg 2013:1–7, 2013

**2**. Dindo D, Demartines N, Clavien PA: Classification of surgical complications: A new proposal with evaluation in a cohort of 6336 patients and results of a survey. Ann Surg 240:205–213, 2004

**3**. Slankamenac K, Graf R, Barkun J, et al: The Comprehensive Complication Index [Internet]. Ann Surg 258:1–7, 2013

**4**. Van Den Broek MAJ, Van Dam RM, Van Breukelen GJP, et al: Development of a composite endpoint for randomized controlled trials in liver surgery [Internet]. Br J Surg 98:1138–1145, 2011[cited 2023 Feb 6]

|  | Patients treated with preoperative chemotherapy, intention-to-treat population | |
| --- | --- | --- |
|  | (n=70) | |
| *Characteristics* | Laparoscopic | Open |
|  | (n=37) | (n=33) |
| Type of preoperative chemotherapy used |  |  |
| Capecitabine | 0 | 2 (6.1) |
| Oxaliplatin | 0 | 1 (3.0) |
| Oxaliplatin/5FU | 2 (5.4) | 1 (3.0) |
| Docetaxel | 0 | 1 (3.0) |
| Paclitaxel | 1 (2.7) | 0 |
| Paclitaxel/Carboplatin | 0 | 1 (3.0) |
| CAPOX | 9 (24.3) | 8 (24.2) |
| CAPOX/Epirubicin | 0 | 1 (3.0) |
| FOLFOX | 12 (32.4) | 10 (30.3) |
| FOLFIRI | 3 (8.1) | 3 (9.1) |
| FOLFIRINOX | 2 (5.4) | 1 (3.0) |
| Unknown | 8 (21.6) | 4 (12.1) |
| Number of cycles | 6 [4, 7] | 6 [4, 8] |
| Values are expressed in counts (percentages) or in median [IQR]. There were no statistically significant differences between the groups. | | |

**Supplementary table 1.** Types of preoperative chemotherapy used and number of cycles in the intention-to-treat population

|  | Intention-to-treat population | |
| --- | --- | --- |
|  | (n=246) | |
| *Characteristics* | Laparoscopic | Open |
|  | (n=122) | (n=124) |
| *Operative characteristics* |  |  |
| Reasons for conversion |  |  |
| Technical difficulties | 8 (47.1%) |  |
| Oncological | 4 (23.5%) |  |
| Bleeding | 3 (17.6%) |  |
| Intolerance of pneumoperitoneum | 2 (11.8%) |  |
| Type of incision |  |  |
| Subcostal right |  | 34 (28.8) |
| Subcostal bilateral |  | 1 (0.8) |
| J-shaped |  | 66 (55.9) |
| Midline/reversed L |  | 17 (14.4) |
| Intraoperative transfusion of packed red blood cells | 12 (10) | 8 (6.6) |
| Pringle manoeuvre applied | 91 (75.8) | 85 (68.5) |
| Total duration of Pringle when applied, minutes | 35.5 [25, 45] | 30 [20, 43.5] |
| *Postoperative endpoints* |  |  |
| Time to reach components of functional recovery, days |  |  |
| Oral analgesics only | 2 [1, 3] | 3 [2, 4] |
| Independently mobile | 3 [2, 4] | 4 [3, 5] |
| Tolerates solid food | 1 [1, 1] | 1 [1, 2] |
| Normal or decreasing laboratory values | 3 [2, 3] | 3 [3, 3.8] |
| Independent of intravenous fluid | 2 [1, 3] | 2 [2, 3] |
| Reasons for delay of discharge after functional recovery |  |  |
| Complications | 4 (10.3) | 12 (21.8) |
| In-hospital administrative reasons | 6 (15.4) | 9 (16.4) |
| Patient does not feel ready | 11 (28.2) | 17 (30.9) |
| Patient transport | 2 (5.1) | 3 (5.5) |
| Post-hospital reasons (e.g. home care arrangements) | 2 (5.1) | 1 (1.8) |
| Unknown | 14 (35.9) | 13 (23.6) |
| Pathological diagnosis |  |  |
| Benign |  |  |
| Hepatocellular adenoma | 1 (0.8) | 4 (3.2) |
| Focal Nodular Hyperplasia | 4 (3.4) | 0 |
| Haemangioma | 3 (2.5) | 1 (0.8) |
| Other benign | 3 (2.5) | 6 (4.8) |
| Malignant |  |  |
| Colorectal metastasis | 67 (56.8) | 62 (50) |
| Non-colorectal metastasis | 5 (4.2) | 6 (4.8) |
| Hepatocellular carcinoma | 28 (23.7) | 39 (31.5) |
| Cholangiocarcinoma | 4 (3.4) | 6 (4.8) |
| Other malignant | 3 (2.5) | 0 |
| Number of lesions in resected specimen(s) |  |  |
| Zero | 0 | 1 (0.8) |
| One | 97 (82.9) | 97 (78.9) |
| Two | 14 (12.0) | 19 (15.4) |
| Three | 4 (3.4) | 5 (4.1) |
| Four | 2 (1.7) | 1 (0.8) |
| Diameter largest lesion in specimen(s), millimetres | 30 [23, 41] | 28 [17.5, 40] |

**Supplementary table 2.** Additional operative and postoperative characteristics of the intention-to-treat population, stratified by the allocated surgical approach

| *Laparoscopic group* | |
| --- | --- |
| Patient one | Underwent a non-anatomical resection in segment 7, the procedure was converted to open due to venous bleeding (intraoperative blood loss 2500 mLs). Intraoperatively the patient received two packed red blood cells. Haemoglobin levels remained stable above 9 mmol/L in the days after the procedure. On postoperative day three the patient had an episode of vomiting on the ward, aspirated and went into cardiac arrest. Resuscitation was unsuccessful. |
| Patient two | Underwent an uncomplicated non-anatomical resection in segment 7 and segment 8 (intraoperative blood loss 800 mLs). The patient initially had an uncomplicated recovery and was discharged on postoperative day five. On postoperative day 16 the patient collapsed, was resuscitated and transferred to the emergency department. A massive pulmonary embolism was diagnosed, and the patient deceased shortly after readmission. |
| Patient three | Underwent an anatomical resection in segment 7, the procedure was converted to open due to adhesions and limited visibility (intraoperative blood loss 1800 mLs). Intraoperatively the patient received six packed red blood cells. The patient developed a prerenal acute kidney injury and received another packed red blood cell on postoperative day one. There were no signs of an active bleeding. Continuous veno-venous hemofiltration was started in the intensive care unit. The patient deceased on postoperative day three due to a cardiac arrest. |
| *Open group* | |
| Patient one | Underwent an uncomplicated anatomical resection in segment 7 (intraoperative blood loss 350 mLs). Postoperatively the patient developed chest pain with a widespread anterolateral and inferior ST-elevation myocardial infarction on the ECG. A coronary angiogram and thrombectomy was performed but the patient remained unstable and deceased. |

**Supplementary table 3.** Clinical course of patients that deceased due to complications

|  | Patients with malignancies, intention-to-treat population | |
| --- | --- | --- |
|  | (n=224) | |
| *Characteristics* | Laparoscopic | Open |
|  | (n=111) | (n=113) |
| Recurrence of disease at follow-up |  |  |
| No | 48 (45.7) | 55 (48.7) |
| Yes | 57 (54.3) | 58 (51.3) |
| Vital status at follow-up |  |  |
| Alive | 79 (73.1) | 86 (76.1) |
| Deceased | 29 (26.9) | 27 (23.9) |
| Cause of death |  |  |
| Postoperative mortality | 2 (6.9) | 1 (3.8) |
| Progressive disease | 17 (58.6) | 15 (57.7) |
| Likely progressive disease | 1 (3.4) | 3 (11.5) |
| Major trauma following car accident | 1 (3.4) | 0 |
| Myocardial infarction | 0 | 1 (3.8) |
| COVID-19 | 1 (3.4) | 0 |
| Hepatic decompensation | 1 (3.4) | 0 |
| Unknown | 6 (20.7) | 6 (23.1) |
| First recurrence site |  |  |
| Cerebral | 2 (3.5) | 0 |
| Colorectal | 1 (1.8) | 0 |
| Liver-only | 28 (49.1) | 37 (63.8) |
| Liver, colorectal | 1 (1.8) | 0 |
| Liver, lung | 6 (10.5) | 5 (8.6) |
| Liver, lymph nodes | 1 (1.8) | 0 |
| Liver, ossal | 3 (5.3) | 1 (1.7) |
| Liver, peritoneum | 0 | 1 (1.7) |
| Liver, lung, adrenal | 1 (1.8) | 0 |
| Liver, lung, lymph nodes | 0 | 1 (1.7) |
| Liver, ossal, peritoneum | 0 | 1 (1.7) |
| Lung | 7 (12.3) | 9 (15.5) |
| Lung, peritoneum | 1 (1.8) | 0 |
| Lymph nodes | 0 | 1 (1.7) |
| Peritoneum | 1 (1.8) | 1 (1.7) |
| Unknown | 3 (5.3) | 1 (1.7) |
| Liver recurrence site in relation to resection margin |  |  |
| Close to resection margin | 3 (7.0) | 5 (10.9) |
| Close and distant to resection margin | 4 (9.3) | 4 (8.7) |
| Distant to resection margin | 35 (81.4) | 36 (78.3) |
| Unknown | 1 (2.3) | 1 (2.2) |
| Values are expressed in counts (percentages).  There were no statistically significant differences between the groups. | | |

**Supplementary table 4.** Oncological outcomes of the patients that underwent surgery for malignancies, intention-to-treat population.

|  | Patients with colorectal liver metastases, intention-to-treat population | |
| --- | --- | --- |
|  | (n=129) | |
| *Characteristics* | Laparoscopic | Open |
|  | (n=67) | (n=62) |
| Resection margin* |  |  |
| R0: resection margin ≥ 1 millimetre | 57 (85.1) | 50 (82) |
| R1: resection margin < 1 millimetre | 9 (13.4) | 11 (18) |
| R2: macroscopically irradical | 1 (1.5) | 0 |
| Recurrence of disease at follow-up |  |  |
| No | 27 (40.3) | 29 (46.8) |
| Yes | 40 (59.7) | 33 (53.2) |
| Vital status at follow-up |  |  |
| Alive | 50 (74.6) | 48 (77.4) |
| Deceased | 17 (25.4) | 14 (22.6) |
| Cause of death |  |  |
| Postoperative mortality | 2 (11.8) | 0 |
| Progressive disease | 7 (41.2) | 6 (42.9) |
| Likely progressive disease | 1 (5.9) | 2 (14.3) |
| Major trauma following car accident | 1 (5.9) | 0 |
| Myocardial infarction | 0 | 1 (7.1) |
| Unknown | 6 (35.3) | 5 (35.7) |
| First recurrence site |  |  |
| Liver-only | 19 (47.5) | 15 (45.5) |
| Liver, colorectal | 1 (2.5) | 0 |
| Liver, lung | 4 (10.0) | 5 (15.2) |
| Liver, lymph nodes | 1 (2.5) | 0 |
| Liver, ossal | 1 (2.5) | 0 |
| Liver, lung, adrenal | 1 (2.5) | 0 |
| Liver, lung, lymph nodes | 0 | 1 (3.0) |
| Liver, ossal, peritoneum | 0 | 1 (3.0) |
| Lung | 7 (17.5) | 9 (27.3) |
| Lung, peritoneum | 1 (2.5) | 0 |
| Lymph nodes | 0 | 1 (3.0) |
| Peritoneum | 1 (2.5) | 1 (3.0) |
| Unknown | 1 (2.5) | 0 |
| Liver recurrence site in relation to resection margin |  |  |
| Close to resection margin | 2 (7.1) | 2 (9.1) |
| Close and distant to resection margin | 4 (14.3) | 2 (9.1) |
| Distant to resection margin | 22 (78.6) | 17 (77.3) |
| Unknown | 0 | 1 (4.5) |
| Values are expressed in counts (percentages).  There were no statistically significant differences between the groups. | | |

**Supplementary table 5.** Oncological outcomes of the patients that underwent surgery for colorectal liver metastases, intention-to-treat population.

|  | Per-protocol population | |
| --- | --- | --- |
|  | (n=235) | |
| *Baseline characteristics* | Laparoscopic | Open |
|  | (n=115) | (n=120) |
| Sex |  |  |
| Male | 77 (67) | 85 (70.8) |
| Female | 38 (33) | 35 (29.2) |
| Age, years | 68 [58, 74] | 68.5 [58.8, 75.3] |
| BMI, kilogram/square meter | 26.7 [23.6, 28.9] | 26.8 [24.2, 29.1] |
| ASA classification |  |  |
| I: healthy | 12 (10.4) | 7 (5.8) |
| II: mild systemic disease | 72 (62.6) | 74 (61.7) |
| III: severe systemic disease | 31 (27.0) | 39 (32.5) |
| ECOG performance status score |  |  |
| O: asymptomatic, normal activity | 88 (76.5) | 92 (76.7) |
| 1: symptomatic, normal activity | 26 (22.6) | 24 (20) |
| 2: symptomatic, <50% bedridden | 1 (0.9) | 4 (3.3) |
| Comorbidity | 82 (71.3) | 92 (76.7) |
| Cardiovascular | 66 (57.4) | 79 (65.8) |
| Respiratory | 15 (13) | 28 (23.3) |
| Other | 5 (4.3) | 7 (5.8) |
| Previous abdominal surgery | 73 (63.5) | 78 (65) |
| Preoperative systemic treatment with chemotherapy | 36 (31.3) | 30 (25) |
| Preoperative diagnosis |  |  |
| Benign | 10 (8.7) | 4 (3.3) |
| Malignant |  |  |
| Colorectal metastasis | 60 (52.2) | 61 (50.8) |
| Non-colorectal metastasis | 7 (6.1) | 7 (5.8) |
| Hepatocellular carcinoma | 31 (27) | 38 (31.7) |
| Cholangiocarcinoma | 4 (3.5) | 4 (3.3) |
| Other | 1 (0.9) | 2 (1.7) |
| Unknown | 2 (1.7) | 4 (3.3) |
| Preoperative size of the largest lesion |  |  |
| <3 centimetres | 62 (53.9) | 64 (53.3) |
| ≥3 centimetres | 53 (46.1) | 56 (46.7) |
| Extrahepatic metastases | 11 (9.6) | 4 (3.3) |
| In the per-protocol analysis, patients in which an attempt to perform a laparoscopic resection required conversion to hand-assisted or open surgery were still included in the laparoscopic group.  Values are expressed in counts (percentages) or in median [IQR].  There were no statistically significant differences between the groups. Abbreviations: BMI, body mass index; ASA, American Society of Anesthesiologists; ECOG, Eastern Cooperative Oncology Group. | | |

**Supplementary table 6.** Baseline characteristics of the per-protocol population, stratified by the allocated surgical approach

|  | Per-protocol population | |
| --- | --- | --- |
|  | (n=235) | |
| *Surgical characteristics* | Laparoscopic | Open |
|  | (n=115) | (n=120) |
| Number of lesion(s) |  |  |
| Single | 100 (87) | 95 (81.9) |
| Multiple | 15 (13) | 21 (18.1) |
| Location of tumour* |  |  |
| Segment 4A | 13 (11.3) | 14 (11.7) |
| Anatomical | 6 (46.2) | 3 (21.4) |
| Non-anatomical | 7 (53.8) | 11 (78.6) |
| Segment 6/7 | 23 (20) | 23 (19.2) |
| Anatomical | 13 (56.5) | 11 (47.8) |
| Non-anatomical | 10 (43.5) | 12 (52.2) |
| Segment 7 | 54 (47) | 36 (30) |
| Anatomical | 19 (35.2) | 12 (33.3) |
| Non-anatomical | 35 (64.8) | 24 (66.7) |
| Segment 8 | 36 (31.3) | 60 (50) |
| Anatomical | 8 (22.2) | 20 (33.3) |
| Non-anatomical | 28 (77.8) | 40 (66.7) |
| Other location | 0 | 0 |
| Additional local treatment |  |  |
| Resection | 4 (3.5) | 6 (5) |
| Ablation | 3 (2.6) | 2 (1.7) |
| Concurrent extrahepatic procedure(s) | 21 (18.3) | 34 (28.3) |
| Cholecystectomy | 18 (85.7) | 29 (85.3) |
| Cholecystectomy, lymphadenectomy | 1 (4.8) | 1 (2.9) |
| Cholecystectomy, open inguinal hernia repair | 1 (4.8) | 0 |
| Lymphadenectomy | 1 (4.8) | 1 (2.9) |
| Incisional hernia repair | 0 | 1 (2.9) |
| Mastectomy, axillary lymph node clearance | 0 | 1 (2.9) |
| Para duodenal lymph node biopsy | 0 | 1 (2.9) |
| In the per-protocol analysis, patients in which an attempt to perform a laparoscopic resection required conversion to hand-assisted or open surgery were still included in the laparoscopic group.  Values are expressed in counts (percentages).  There were no statistically significant differences between the groups. *Counts exceed the sample because some patients had multiple tumours, type of resection only specified when performed | | |

**Supplementary table 7.** Surgical characteristics of the per-protocol population, stratified by the allocated surgical approach

|  | Per-protocol population | | Unadjusted difference (96% CI) | P | Adjusted difference (96% CI) | P |
| --- | --- | --- | --- | --- | --- | --- |
|  | (n=235) | |  |  |  |  |
|  | Laparoscopic | Open |  |  |  |  |
|  | (n=115) | (n=120) |  |  |  |  |
| *Primary endpoint* | | | | | | |
| Time to functional recovery, days | 3 [3, 5] | 4 [3, 5] | -18.4% (-28.3% to -7.3%) | 0.001 | -21.1% (-30.8% to -10.2%) | <0.001 |
| *Secondary endpoints* | | | Unadjusted difference (99% CI) | P | Adjusted difference (99% CI) | P |
| **Intraoperative** | | | | | | |
| Operation duration, minutes | 240 [190.8, 300] | 200 [156.3, 270] | 22.5% (10.5% to 35.7%) | <0.001 | 22.2% (10.8% to 34.7%) | <0.001 |
| Estimated blood loss, millilitres | 200 [100, 475] | 250 [100, 400] | 18.8% (-13.3% to 62.9%) | 0.158 | 21% (-10.6% to 63.8%) | 0.104 |
| Unfavourable intraoperative incidents |  |  | OR 1.45 (0.48 to 4.37) | 0.380 | aOR 1.63 (0.52 to 5.15) | 0.271 |
| Satava 1 | 11 (9.6) | 13 (10.8) |  |  |  |  |
| Satava 2 | 5 (4.3) | 0 |  |  |  |  |
| Satava 3 | 1 (0.9) | 0 |  |  |  |  |
| Conversion |  |  |  |  |  |  |
| To a hand-assisted procedure | 1 (0.9) |  |  |  |  |  |
| To an open procedure | 14 (12.2) |  |  |  |  |  |
| **Postoperative** | | | | | | |
| Postoperative length of stay, days | 4 [3, 5] | 5 [4, 7] | -23% (-33.5% to 10.9%) | <0.001 | -22.9% (-10.8% to -33.4%) | <0.001 |
| 90-day overall morbidity | 17 (14.9) | 28 (23.9) | OR 0.51 (0.20 to 1.34) | 0.073 | aOR 0.49 (0.18 to 1.33) | 0.065 |
| Mild (Clavien-Dindo grade I or II) | 11 (9.6) | 17 (14.5) | OR 0.54 (0.16 to 1.87) | 0.201 | aOR 0.50 (0.14 to 1.76) | 0.153 |
| Severe (Clavien-Dindo grade ≥ IIIA) | 6 (5.3) | 11 (9.4) | OR 0.54 (0.14 to 2.17) | 0.256 | aOR 0.54 (0.13 to 2.26) | 0.267 |
| Liver-specific | 7 (6.1) | 14 (12.0) | OR 0.48 (0.14 to 1.68) | 0.132 | aOR 0.49 (0.14 to 1.77) | 0.154 |
| 90-day readmission | 7 (6.2) | 12 (10.3) | OR 0.57 (0.15 to 2.11) | 0.265 | aOR 0.56 (0.15 to 2.12) | 0.258 |
| 90-day or in-hospital mortality | 3 (2.6) | 1 (0.9) | OR 3.58 (0.17 to 74.64) | 0.278 | aOR 3.08 (0.15 to 64.86) | 0.342 |
| Complication-related | 3 (2.6) | 1 (0.9) |  |  |  |  |
| Comprehensive Complication Index |  |  | OR 0.54 (0.19 to 1.53) | 0.125 | aOR 0.55 (0.20 to 1.53) | 0.131 |
| Category A (Score 0-20) | 102 (89.5) | 96 (82.1) |  |  |  |  |
| Category B (Score 20-60) | 9 (7.9) | 20 (17.1) |  |  |  |  |
| Category C (Score 60-100) | 3 (2.6) | 1 (0.9) |  |  |  |  |
| Delay of discharge after functional recovery | 36 (32.4) | 54 (45.8) | OR 0.49 (0.21 to 1.11) | 0.025 | aOR 0.47 (0.20 to 1.09) | 0.020 |
| Resection margin** |  |  | OR 0.73 (0.24 to 2.25) | 0.471 | aOR 0.78 (0.25 to 2.44) | 0.568 |
| R0: resection margin ≥ 1 millimetre | 91 (87.5) | 93 (85.3) |  |  |  |  |
| R1: resection margin < 1 millimetre | 12 (11.5) | 15 (13.8) |  |  |  |  |
| R2: macroscopically irradical | 1 (1) | 1 (0.9) |  |  |  |  |
| Time to initiation of adjuvant chemotherapy, days ¥ | 44 [35.3, 62.3] | 62 [46.5, 73.5] | HR 1.40 (0.60 to 3.25) | 0.300 | aHR 1.16 (0.47 to 2.83) | 0.670 |
| Incisional hernia at one year follow-up | 5 (7.9) | 5 (6.2) | OR 1.13 (0.131 to 9.73) | 0.884 | aOR 0.51 (0.03 to 7.59) | 0.524 |
| In the per-protocol analysis, patients in which an attempt to perform a laparoscopic resection required conversion to hand-assisted or open surgery were still included in the laparoscopic group.  Values are expressed in counts (percentages) or in median [IQR].  Abbreviations: CI, confidence interval; OR, odds ratio; aOR, adjusted odds ratio.  *The open group was consistently used as reference group.  ⌑Adjusted for sex, age, benign/malignant lesion type, and centre.  **In case of malignancy  ¥When treated with adjuvant chemotherapy, patients that underwent a subsequent resection of the primary and received chemotherapy after this procedure were excluded. | | | | | | |

**Supplementary table 8.** Primary and secondary endpoint analyses of the per-protocol population, stratified by the allocated surgical approach

|  | Patients with malignancies, per-protocol population | |
| --- | --- | --- |
|  | (n=209) | |
| *Characteristics* | Laparoscopic | Open |
|  | (n=104) | (n=109) |
| Recurrence of disease at follow-up |  |  |
| No | 47 (46.1) | 53 (48.6) |
| Yes | 55 (53.9) | 56 (51.4) |
| Vital status at follow-up |  |  |
| Alive | 77 (75.5) | 83 (76.1) |
| Deceased | 25 (24.5) | 26 (23.9) |
| Cause of death |  |  |
| Postoperative mortality | 2 (8.0) | 1 (4.0) |
| Progressive disease | 14 (56.0) | 15 (60.0) |
| Likely progressive disease | 1 (4.0) | 3 (12.0) |
| Major trauma following car accident | 1 (4.0) | 0 |
| Myocardial infarction | 0 | 1 (4.0) |
| COVID-19 | 1 (4.0) | 0 |
| Hepatic decompensation | 1 (4.0) | 0 |
| Unknown | 5 (20.0) | 5 (20.0) |
| First recurrence site |  |  |
| Cerebral | 2 (3.6) | 0 |
| Colorectal | 1 (1.8) | 0 |
| Liver-only | 27 (49.1) | 36 (64.3) |
| Liver, colorectal | 1 (1.8) | 0 |
| Liver, lung | 5 (9.1) | 5 (8.9) |
| Liver, lymph nodes | 1 (1.8) | 0 |
| Liver, ossal | 3 (5.5) | 1 (1.8) |
| Liver, peritoneum | 0 | 1 (1.8) |
| Liver, lung, adrenal | 1 (1.8) | 0 |
| Liver, lung, lymph nodes | 0 | 1 (1.8) |
| Liver, ossal, peritoneum | 0 | 1 (1.8) |
| Lung | 7 (12.7) | 8 (14.3) |
| Lung, peritoneum | 1 (1.8) | 0 |
| Lymph nodes | 0 | 1 (1.8) |
| Peritoneum | 1 (1.8) | 1 (1.8) |
| Unknown | 3 (5.5) | 1 (1.8) |
| Liver recurrence site in relation to resection margin |  |  |
| Close to resection margin | 2 (4.9) | 5 (11.1) |
| Close and distant to resection margin | 4 (9.8) | 4 (8.9) |
| Distant to resection margin | 34 (82.9) | 35 (77.8) |
| Unknown | 1 (2.4) | 1 (2.2) |
| Values are expressed in counts (percentages). There were no statistically significant differences between the groups. | | |

**Supplementary table 9.** Oncological outcomes of the patients that underwent surgery for malignancies, per-protocol population.

|  | Patients with colorectal liver metastases, per-protocol population | |
| --- | --- | --- |
|  | (n=125) | |
| *Characteristics* | Laparoscopic | Open |
|  | (n=66) | (n=59) |
| Resection margin* |  |  |
| R0: resection margin ≥ 1 millimetre | 56 (84.8) | 48 (81.4) |
| R1: resection margin < 1 millimetre | 9 (13.7) | 11 (18.6) |
| R2: macroscopically irradical | 1 (1.5) | 0 |
| Recurrence of disease at follow-up |  |  |
| No | 27 (40.9) | 28 (47.5) |
| Yes | 39 (59.1) | 31 (52.5) |
| Vital status at follow-up |  |  |
| Alive | 50 (75.8) | 46 (78) |
| Deceased | 16 (24.2) | 13 (22) |
| Cause of death |  |  |
| Postoperative mortality | 2 (12.5) | 0 |
| Progressive disease | 7 (43.8) | 6 (46.2) |
| Likely progressive disease | 1 (6.2) | 2 (15.4) |
| Major trauma following car accident | 1 (6.2) | 0 |
| Myocardial infarction | 0 | 1 (7.7) |
| Unknown | 5 (31.2) | 4 (30.8) |
| First recurrence site |  |  |
| Liver-only | 19 (48.7) | 14 (45.2) |
| Liver, colorectal | 1 (2.6) | 0 |
| Liver, lung | 3 (7.7) | 5 (16.1) |
| Liver, lymph nodes | 1 (2.6) | 0 |
| Liver, ossal | 1 (2.6) | 0 |
| Liver, lung, adrenal | 1 (2.6) | 0 |
| Liver, lung, lymph nodes | 0 | 1 (3.2) |
| Liver, ossal, peritoneum | 0 | 1 (3.2) |
| Lung | 7 (17.9) | 8 (25.8) |
| Lung, peritoneum | 1 (2.6) | 0 |
| Lymph nodes | 0 | 1 (3.2) |
| Peritoneum | 1 (2.6) | 1 (3.2) |
| Unknown | 1 (2.6) | 0 |
| Liver recurrence site in relation to resection margin |  |  |
| Close to resection margin | 2 (7.4) | 2 (9.5) |
| Close and distant to resection margin | 4 (14.8) | 2 (9.5) |
| Distant to resection margin | 21 (77.8) | 16 (76.2) |
| Unknown | 0 | 1 (4.8) |
| Values are expressed in counts (percentages).  There were no statistically significant differences between the groups. | | |

**Supplementary table 10.** Oncological outcomes of the patients that underwent surgery for colorectal liver metastases, per-protocol population.

|  | Intention-to-treat population | |
| --- | --- | --- |
|  | (n=246) | |
| *Characteristics* | Laparoscopic | Open |
|  | (n=122) | (n=124) |
| Time to functional recovery, days | 2 (1.6) | 1 (0.8) |
| Sex | 0 | 0 |
| Age, years | 0 | 0 |
| BMI, kilogram/square metre | 1 (0.8) | 0 |
| ASA classification | 0 | 0 |
| ECOG performance status score | 0 | 0 |
| Comorbidity | 0 | 0 |
| Previous abdominal surgery | 0 | 0 |
| Preoperative systemic treatment with chemotherapy | 0 | 0 |
| Preoperative diagnosis | 0 | 0 |
| Preoperative size of the largest lesion | 0 | 0 |
| Extrahepatic metastases | 0 | 0 |
| Number of tumour(s) | 2 (1.6) | 4 (3.2) |
| Location of resection | 0 | 0 |
| Additional local treatment | 0 | 0 |
| Concurrent extrahepatic procedures | 0 | 0 |
| Operation duration, minutes | 4 (3.3) | 3 (2.4) |
| Estimated blood loss, millilitres | 1 (0.8) | 1 (0.8) |
| Unfavourable intraoperative incidents | 1 (0.8) | 0 |
| Conversion | 0 | NA |
| Postoperative length of stay, days | 3 (2.5) | 1 (0.8) |
| 90-day overall morbidity | 2 (1.6) | 4 (3.2) |
| 90-day readmission | 2 (1.6) | 4 (3.2) |
| 90-day or in-hospital mortality | 2 (1.6) | 4 (3.2) |
| Comprehensive Complication Index | 2 (1.6) | 4 (3.2) |
| Resection margin | 5 (4.1) | 0 |
| Time to initiation of adjuvant chemotherapy, days | 1 (0.8) | 1 (0.8) |
| Incisional hernia at one year follow-up | 57 (46.7) | 41 (33.1) |
| Reasons for conversion | 0 | NA |
| Type of incision | NA | 6 (4.8) |
| Intraoperative transfusion of packed red blood cells | 2 (1.6) | 2 (1.6) |
| Pringle manoeuvre applied | 2 (1.6) | 0 |
| Total duration of Pringle when applied, minutes | 0 | 0 |
| Delay in discharge after functional recovery | 6 (4.9) | 2 (1.6) |
| Pathological diagnosis | 4 (3.3) | 0 |
| Number of lesions in resected specimen(s) | 5 (4.1) | 1 (0.8) |
| Diameter largest tumour in specimen(s), millimetres | 5 (4.1) | 1 (0.8) |

**Supplementary table 11.** Missing data

| Country | Centre |
| --- | --- |
| Belgium | Groeninge Hospital |
| Italy | Fondazione Poliambulanza Istituto Ospedaliero |
|  | IRCCS San Raffaele Hospital |
|  | Federico II University Hospital |
|  | San Camillo Hospital |
| Norway | Oslo University Hospital |
| Russia | Moscow Clinical Research Centre |
| The Netherlands | Amsterdam UMC location University of Amsterdam |
|  | Maastricht University Medical Centre+ |
| United Kingdom | University Hospital Southampton NHS Foundation Trust |
|  | Aintree University Hospital |
|  | Manchester University Foundation trust |
|  | King's College Hospital |
|  | Oxford University Hospital NHS Foundation Trust |
|  | University Hospitals Plymouth NHS Trust |
|  | Newcastle upon Tyne Hospitals NHS Foundation Trust |
|  | University Hospitals Birmingham NHS Foundation Trust |

**Supplementary table 12.** Participating centres per country

|  | Intention-to-treat population | | ICC |
| --- | --- | --- | --- |
|  | (n=246) | |  |
|  | Laparoscopic | Open |  |
|  | (n=122) | (n=124) |  |
| Time to functional recovery, days | 3 [3, 5] | 4 [3, 5] | 0 |
| *Secondary endpoints* | | |  |
| Operation duration, minutes | 240 [186.3, 300] | 200 [155, 270] | 0.02 |
| Estimated blood loss, milliliters | 200 [100, 500] | 250 [100, 400] | 0 |
| Unfavorable intraoperative incidents |  |  | 0 |
| Satava 1 | 12 (9.9) | 13 (10.5) |  |
| Satava 2 | 6 (5) | 0 |  |
| Satava 3 | 1 (0.8) | 0 |  |
| Conversion |  |  |  |
| To a hand-assisted procedure | 1 (0.8) |  |  |
| To an open procedure | 16 (13.1) |  |  |
| Postoperative length of stay, days | 4 [3, 5] | 5 [4, 7] | 0 |
| 90-day overall morbidity | 17 (14.2) | 28 (23.3) | 0 |
| Mild (Clavien-Dindo grade I or II) | 11 (9.2) | 17 (14.2) |  |
| Severe (Clavien-Dindo grade ≥ IIIA) | 6 (5) | 11 (9.2) |  |
| Liver-specific | 7 (5.8) | 14 (11.7) |  |
| 90-day readmission | 7 (5.9) | 12 (10.1) | 0.14 |
| 90-day or in-hospital mortality | 5 (4.2) | 1 (0.8) | 0 |
| Complication-related | 3 (2.5) | 1 (0.8) | 0 |
| Disease-related | 2 (1.7) | 0 |  |
| Comprehensive Complication Index |  |  | 0.02 |
| Category A (Score 0-20) | 108 (90) | 99 (82.5) |  |
| Category B (Score 20-60) | 9 (7.5) | 20 (16.7) |  |
| Category C (Score 60-100) | 3 (2.5) | 1 (0.8) |  |
| Delay of discharge after functional recovery | 39 (33.6) | 55 (45.1) | 0 |
| Resection margin |  |  | 0 |
| R0: resection margin ≥ 1 millimeter | 93 (87.7) | 97 (85.8) |  |
| R1: resection margin < 1 millimeter | 12 (11.3) | 15 (13.3) |  |
| R2: macroscopically irradical | 1 (0.9) | 1 (0.9) |  |
| Time to initiation of adjuvant chemotherapy, days | 44 [35, 60.8] | 61 [45.8, 72.8] | 0 |
| Incisional hernia at one year follow-up | 6 (9.2) | 5 (6) | 0 |

**Supplementary table 13.** Intracluster correlation coefficients (ICC) of the participating centres
