# Supplementary material for: Laparoscopic versus open parenchymal preserving liver resections in the posterosuperior segments (ORANGE Segments): a multicentre, single-blind, randomised controlled trial
Source: Lancet Reg Health Eur. 2025 Feb 20;51:101228. doi: 10.1016/j.lanepe.2025.101228 (PMC11889631; doi:10.1016/j.lanepe.2025.101228)
Supplement: 1 - DSMB charter [file mmc3.pdf]

DSMB – Charter

ORANGE II PLUS -Trial: An international multicentre randomised controlled trial of optimised surgical recovery after open versus laparoscopic hemihepatectomy.

**Principal investigators:**<br><br>**Prof. C.H.C. Dejong, MD, PhD**<br>**Surgeon**	**R.M. van Dam, MD**<br>**Surgeon**
Phone: +31 43 387 65 43 Pager: 6256<br>Email: r.vandam@orangetrial.eu	Phone: +31 43 387 65 43
**Study Coordinator / Investigator:**<br><br>**E. M. Wong-Lun-Hing, MD**	
Phone: +31 43 387 65 43 Pager: 9036<br>Email: e.wong@orangetrial.eu	
**Address:**<br>Department of Surgery<br>Maastricht University Medical Centre<br>PO Box 5800<br>6202 AZ Maastricht<br>The Netherlands	
**Funding:**<br><br>None	**Registration:**<br><br>www.ClinicalTrials.gov: NCT 01441856

1. INTRODUCTION

This charter is for the Data Safety and Monitoring Board (DSMB) of the ***ORANGE II PLUS - Trial: An international multicentre randomised controlled trial of optimised surgical recovery after open versus laparoscopic hemihepatectomy.***

The objective of this trial is to determine in patients undergoing a hemihepatectomy (left or right) whether laparoscopic surgery compared to open surgery further ads to the speed of recovery within an enhanced recovery programme. The ORANGE-II PLUS trial is a prospective study with an experimental design, that produces two double-blinded randomized arms and a prospective registry to determine whether laparoscopic surgery is to be preferred over open surgery in patients undergoing a hemihepatectomy and participating in an enhanced recovery programme.

2. ROLES AND RESPONSIBILITIES

The DSMB Chair has the overall responsibility for the chairing of the DSMB committee and reporting its recommendations. The DSMB functions as a committee within the ORANGE II PLUS – Trial organisation. The charge to the DSMB is to safeguard the interests of trial participants, assess the safety and efficacy of the interventions during the trial, and monitor the overall conduct of the clinical trial.

<u>DSMB Functions and Activities</u>

The DSMB receives and reviews the progress of accruing data of this trial and provides advice on the conduct of the trial to the Principal Investigator. The DSMB informs the Principal Investigator if, in their view:

I. the results are likely to convince a broad range of clinicians, including those supporting the trial and the general clinical community, that one trial arm is clearly indicated or contraindicated, and there was a reasonable expectation that this new evidence would materially influence patient management; or
II. it becomes evident that no clear outcome would be obtained.

In reference to the Damocles Study Group (*Lancet 2005*) the following goals are formulated:

I. Guarantee of patient safety
II. Vigilance of quality of performance within the trial (trial conduct)
III. Stopping rules evaluation (superior / inferior effect)

3. BEFORE OR EARLY IN THE TRIAL

All potential DSMB members will have sight of the protocol before agreeing to join the committee. If a potential DSMB member has major reservations about the trial (e.g. the protocol or the logistics) they should report these to the Principal Investigator and may decide not to accept the invitation to join. DSMB members should be independent and constructively critical of the ongoing trial, but also supportive of aims and methods of the trial. Members of a DSMB will have to sign a contract making clear the need for confidentiality and the liability status of the DSMB members.

4. COMPOSITION

The Board has an advisory role and consists of a chair, a statistician and a surgeon. Committee members serve for the duration of the trial. All the DSMB members will receive reimbursement of travel and accommodation expenses. The DSMB members have disclosed all competing interests to enhance credibility (See Appendix 1).

The members of the DSMB for this trial are:

(1) Prof. M.A. Boermeester, MD, PhD (Chair)
(2) Prof. I.H.M. Borel Rinkes, MD, PhD (Member)
(3) Prof. M. Prins, PhD (Independent statistician)

The Chair

Professor M.A. Boermeester was approached as the chair, for she specializes in benign HPB surgery and abdominal infections and she has gained wide experience as a trial / project leader. She will summarise discussions of DSMB consultations, and is the primary spokesperson for the DSMB.

The independent statistician

The independent statistician, professor M. Prins, will review the report of the Trial Coordinator and his team to the DSMB and will participate in DSMB discussions.

The Principal Investigators

The PI, may be asked, and should be available, to attend open sessions of the DSMB meeting. The other trial members will not usually be expected to attend, but can attend open sessions when necessary (See Organisation of DSMB Meetings).

Secretarial services will be provided by Mrs. Roquet or Mr. E.M. Wong-Lun-Hing, MD (Trial Coordinator)

5. ORGANISATION OF DSMB COUNSELING

The DSMB will evaluate the review report after every 50 (randomized) patients, a total sample size of 250 randomized patients is required, to evaluate the ORANGE II PLUS – Trial's progress, data and safety records. Meetings can be scheduled for further discussion or review of additional data, depending on the reported data in a DSMB report.

6. TRIAL DOCUMENTATION AND PROCEDURES TO ENSURE CONFIDENTIALITY AND PROPER COMMUNICATION

Intended content of material to be available in closed sessions:

Only the DSMB members will not be blinded for the treatment allocation, and the reports provided by E.M. Wong-Lun-Hing and the trial statistician will consist of:

1. After each 50 included patients, within 3 weeks (independent of all 50 have completed follow-up):
   - Short description of the trial's progress, including:
     - Number of randomised patients
     - Number of included patients per site
     - Some baseline characteristics of the randomised patients per intervention
     - Length of hospital stay
     - Percentage of readmission
   - An overview of all (serious) adverse events per arm:
     - Every death will be studied by the DMSB for possible causes and relation with the intervention / study. In addition, every study related serious adverse event will be reported to the Ethical Committee (METC).
     - (Serious) adverse events will also be reported to the Ethical Committee (METC) and a safety report will be constructed annually.

   If needed the DSMB may ask for further, clarifying data.

2. After randomisation and completion of follow-up of 50% of the total needed randomised patients:
   - A sample size of 2 x 125 patients in the randomisation arms can demonstrate a 2 day reduction with a level of significance α=0.04 and a power of 80%.We have chosen to use two simple and transparent stopping rules during the interim analysis:
     1) Stopping for significance

To perform an interim analysis after 50% of the needed randomized sample size (N = 125) has been included, we will use a two-tailed alpha of 0.01 for the primary outcome. This will lead to early stopping upon significance. The trial will be continued if the alfa is greater than 0.01 with a power of 42%.

2) <u>Stopping for safety</u>
The trial will be stopped if the mortality after hemihepatectomy in patients with a normal liver is greater than 5% or is greater than 10% in cirrhotic patients at interim analysis after 50% of the needed randomized sample size (N = 125) has been included.

<u>Access to results</u>
All DSMB members, the Trial Statistician and Trial Coordinator will have access to the accumulating data and interim analysis. They do not have the right to share confidential information with anyone outside the DSMB, including the PI.

<u>External evidence:</u>
Identification and circulation of external evidence (e.g. from other trials/ systematic reviews) is not the responsibility of the DSMB members. The Principal Investigator or the Trials Coordinator will collate any such information.

<u>Recommendations:</u>
The DSMB will report its recommendations in writing to the Principal Investigator.

<u>Confidential papers:</u>
The DSMB members should store the papers safely after each meeting so they may check the next report against them. After the trial is reported, the DSMB members should destroy all interim reports.

7. RECOMMENDATIONS

The members of the DSMB will provide recommendations based on consensus. If no consensus can be reached after deliberations, the opinion of majority within the DSMB will be decisive. Hence, recommendations can only be made if all three members have given their opinion. It is important that the implications (e.g. ethical, statistical, practical, and financial) for the trial be considered before any recommendation is made.

The DSMB will have the following possible recommendations to make:

- No action is needed, trial continues as planned

- Early stopping due, for example, to clear benefit or harm of a treatment, futility, or external evidence
- Extending recruitment or extending follow-up
- Sanctioning and/or proposing protocol changes

The primary endpoint and the trial's safety and/or efficacy should be the leading factors for the DSMB members' recommendations. In addition, the ORANGE II PLUS – Trial is based on a design with two randomized arms and a prospective registry, in which the patient accrual may differ. Significant differences in patient accrual between the arms and results after interim analysis should be taken into account before reaching any recommendations.

8. REPORTING

- The DSMB will report a recommendation within 3 weeks after their meeting. The Board will report their findings to the Principal Investigator.
- The reports will hold at least a recommendation, preferably accompanied by a summary of the main arguments on which the consensus was reached. The individual opinion of each DSMB member should not be mentioned in these reports.
- If the Principal Investigator disagrees with the reporting an open session should be organised to discuss the different opinion, until a consensus is reached.

9. AFTER THE TRIAL

<u>Publications of results:</u>

At the end of the trial there will be a meeting to allow the DSMB to discuss the final data with principal trial investigators / sponsors and give advice about data interpretation. The DSMB members will be named and their affiliations listed in the main report, unless they explicitly requested otherwise. A brief summary of the timings and conclusions of DSMB meetings will be included in the body of this paper.

The members of the DSMB may discuss issues from their involvement in the trial after publication of the primary trial results, or when the Principal Investigator has given permission.

10. APPENDICES

- Appendix 1: Figure summarising trial
- Appendix 2: Competing interest form

Appendix 1: Trial flowchart

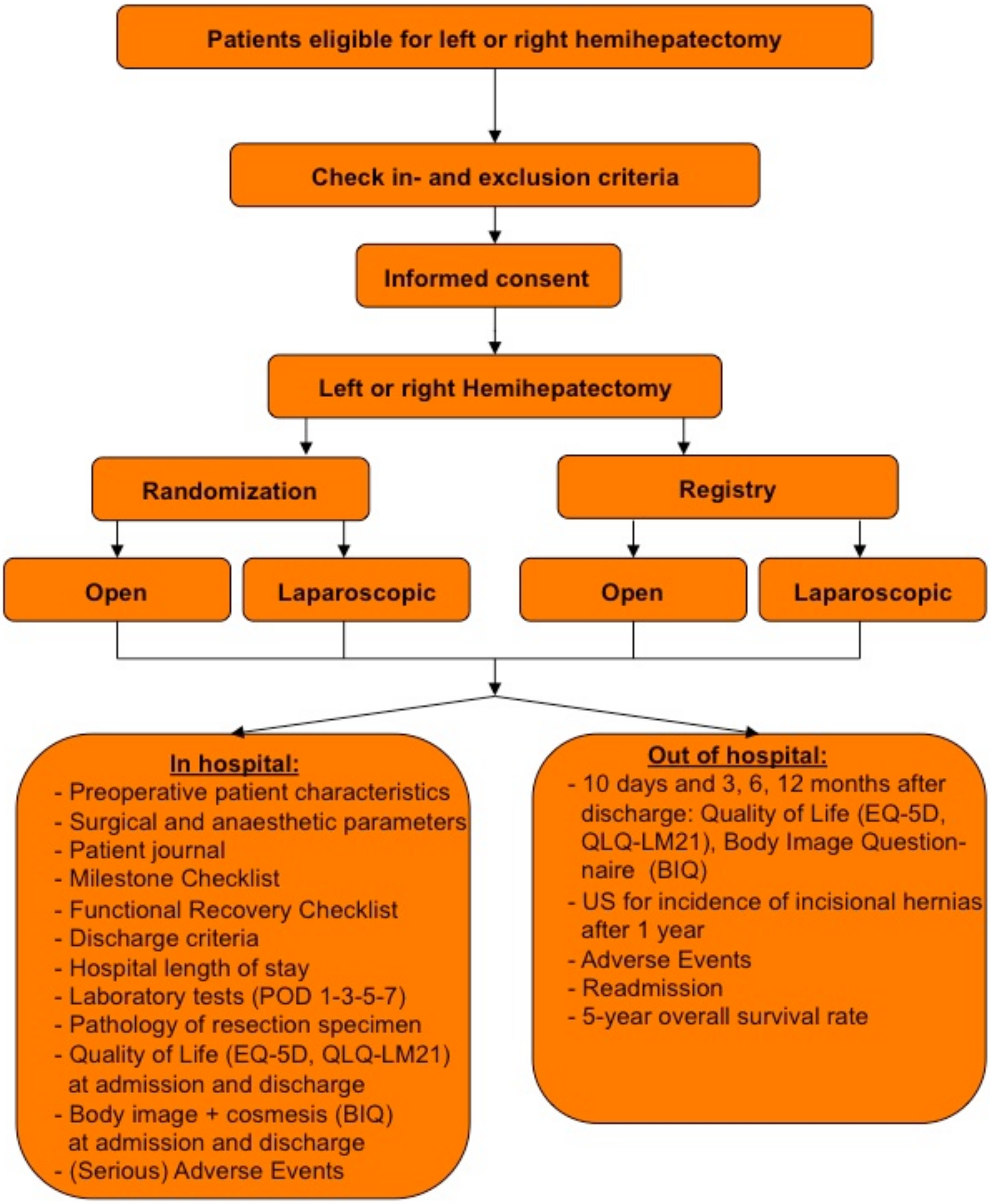

Appendix 2: Competing interests form

Potential competing interests of Data Monitoring Committee members for "*The ORANGE II PLUS - Trial: an international multicentre randomised controlled trial of optimised surgical recovery after open versus laparoscopic surgery within an enhanced recovery ERAS® programme.*"

The avoidance of any perception that members of a DSMB may be biased in some fashion is important for the credibility of the decisions made by the DSMB and for the integrity of the trial.

Possible competing interest should be disclosed via the trials office. In many cases simple disclosure up front should be sufficient. Otherwise, the (potential) DSMB member should remove the conflict or stop participating in the DSMB. Table 1 lists potential competing interests.

Table 1: Potential competing interests

- Stock ownership in any commercial companies involved
- Stock transaction in any commercial company involved (if previously holding stock)
- Consulting arrangements with the sponsor
- Frequent speaking engagements on behalf of the intervention
- Career tied up in a product or technique assessed by trial
- Hands-on participation in the trial
- Involvement in the running of the trial
- Emotional involvement in the trial
- Intellectual conflict e.g. strong prior belief in the trial's experimental arm
- Involvement in regulatory issues relevant to the trial procedures
- Investment (financial or intellectual) in competing products
- Involvement in the publication

Please complete the following section and return to the trials office.

☐ **No,** I have no competing interests to declare
☐ **Yes,** I have competing interests to declare (please detail below)

Please provide details of any competing interests: ______________________________

____

____

Name: ____________________________

Signed: ____________________________ Date: _______________
